# Residential green environments are associated with human milk oligosaccharide diversity and composition

**DOI:** 10.1038/s41598-022-27317-1

**Published:** 2023-01-05

**Authors:** Mirkka Lahdenperä, Laura Galante, Carlos Gonzales-Inca, Jussi Vahtera, Jaana Pentti, Samuli Rautava, Niina Käyhkö, Chloe Yonemitsu, Julia Gupta, Lars Bode, Hanna Lagström

**Affiliations:** 1 Department of Biology, University of Turku, Turku, Finland; 2 Department of Public Health, University of Turku and Turku University Hospital, Turku, Finland; 3 Centre for Population Health Research, University of Turku and Turku University Hospital, Turku, Finland; 4 School of Health and Social Care, Faculty of Medicine, Health and Life Sciences, Swansea University, Swansea, SA2 8PP UK; 5 Department of Geography and Geology, University of Turku, Turku, Finland; 6 Clinicum, Faculty of Medicine, University of Helsinki, Helsinki, Finland; 7 Department of Pediatrics, University of Turku, Turku, Finland; 8 Children’s Hospital, University of Helsinki and Helsinki University Hospital, Helsinki, Finland; 9 Department of Pediatrics and Larsson-Rosenquist Foundation Mother-Milk-Infant Center of Research Excellence (MOMI CORE), University of California San Diego, La Jolla, CA USA

**Keywords:** Nutrition, Paediatric research, Environmental impact, Biodiversity, Immune tolerance

## Abstract

Increased exposure to greener environments has been suggested to lead to health benefits in children, but the associated mechanisms in early life, particularly via biological mediators such as altered maternal milk composition, remain largely unexplored. We investigated the associations between properties of the mother’s residential green environment, measured as (1) greenness (Normalized Difference Vegetation index, NDVI), (2) Vegetation Cover Diversity (VCDI) and (3) Naturalness Index (NI), and human milk oligosaccharides (HMOs), known for their immune- and microbiota-related health effects on the infant (N = 795 mothers). We show that HMO diversity increases and concentrations of several individual HMOs and HMO groups change with increased VCDI and NI in residential green environments. This suggests that variation in residential green environments may influence the infant via maternal milk through modified HMO composition. The results emphasize the mediating role of breastfeeding between the residential green environments and health in early life.

## Introduction

Humans are increasingly disconnected from nature, partly due to the global rise in urbanization and drastic changes in lifestyles^1^. Still, human health is strongly impacted by the environment we live in^2^. Particularly, the quality of residential green spaces are associated with health outcomes already during the prenatal period and in early life, including reduced risk of pre-term birth^3^ and obesity^4^, improved cognitive development^5^ and decreased risks for disease such as atopic sensitization^6^, respiratory diseases^7^ and psychiatric disorders^8^. Likewise, the residential green living environment has been linked to maternal health, including better mental health^3^, lower risk of cardio-vascular diseases and death^9,10^. While we know that green environments play a role in child and maternal health, much remains to be known about the mechanisms through which various characteristics of green spaces impact on health outcomes. Particularly, the mechanisms that function during early life, the period that significantly determines later health^11^, are largely unknown. These mechanisms are potentially complex, and might be mediated by direct physical or airborne contact with the natural environment and by indirect effects via maternal care and breastfeeding^12,13^.

Maternal milk is the ideal nutrition for term, healthy infants and the benefits of breastfeeding on child short- and long-term health are numerous^14,15^. Human milk has evolved to promote survival and healthy development of the infant both through its nutritional composition^16^ and with non-nutritive bioactive factors such as hormones^16,17^, growth factors^16,17^, antibodies^17,18^ and human milk oligosaccharides (HMOs^19,20^). These compounds are also likely to act as chemical messengers informing the infant about maternal and environmental conditions. HMOs are strong candidates for having specific links with the residential green environment, due to the suggested role of environmental microbiota in their composition^21^ and their known modulatory effects on infant health and development. HMOs are a group of more than 150 different complex carbohydrates that together constitute the third largest component in human milk^19,22^. The total amount and composition, however, varies largely between women, and is driven by fixed maternal genetics^19^ (e.g. secretor status) as well as modifiable factors^22^ such as maternal nutrition and exercise. HMO composition has been also associated with wider scale environmental measures such as geographical areas and seasons^23–25^. HMOs have several important health-related functions for the infant. These include shaping the development of the gut microbiota by promoting the growth of specific microbes, preventing pathogen invasion and attachment, and altering immune cell responses, possibly in different sites throughout the body and not only in the infant gut^19,20^. HMOs may thus decrease childhood risk of infections, allergies, auto-immune diseases and inflammation^20^. Similarly, contact with green environments has been suggested to promote immune balance and protect from allergies and inflammatory disorders in childhood through enriching the human skin and gut microbiota, and the evidence for this association is accumulating rapidly^26–29^. It has been specifically hypothesized that the key characteristics driving these associations are the microbial diversity, plant species diversity and abundance, and properties of green land cover types around living environments (e.g. proportion of green space, types of vegetation, and degree of human impact on nature^10,26,30^). Despite the strong immunity-modifying health effects of both HMOs and green living environment, the potential associations between HMOs and properties of the residential green environment have remained unexplored.

The aim of this study was to investigate associations between HMO composition and the ecological properties of the green environments surrounding the homes of the participants (herein referred to as residential green environment) of a Finnish cohort of newborn babies and their mothers (the STEPS Study^31^). We hypothesized that the greater the diversity and proportion of the green environments in the residential area of each mother, the greater are HMO diversity and HMO concentrations. We thus expect that in an environment where we might have more microbial variability the milk would be richer in immunological compounds^32^, in this case HMOs. The residential data is explicit in space and time, including information on the surroundings of the homes of children and their parents in Southwest Finland, allowing detailed level investigation between the HMOs and green environment properties. We established the following residential green environment properties, previously connected to health^33,34^ and microbiota levels^35^: greenness (Normalized Difference Vegetation index, NDVI)^36^, Vegetation Cover Diversity (Simpson's Diversity Index of Vegetation Cover, VCDI)^37^, and Naturalness Index i.e. how much human impact and intervention there has been in the residential area (NI)^38^. The residential variables were measured at the time of child was born (2008–2010) with 750 m × 750 m grid size around the homes of the families, capturing better the variation of green environment properties associated with childhood immunity related diseases than smaller grids in Finland^39^, and as microbes might also be transmitted via pollen grains, dust, and ambient air, travelling large distances^28^. The milk samples were collected around the child age of 3 months and investigated for HMO composition (diversity of HMOs, total sum of HMOs and concentrations of HMO-bound sialic acid, HMO-bound fucose, 19 most abundant individual HMOs (constituting ~ 90% of all HMOs) and seven HMO groups based on structural features). The analyses were adjusted for a wide array of individual level maternal and child characteristics in line with previous studies^22^ and residential socioeconomic disadvantage. Exploring the associations between residential green environments and HMOs is a critical first step toward understanding the mechanistic pathways of natural environments on child health in early life, also emphasizing the role of green environments as an immune function modifier in childhood.

## Results

### Mother and infant characteristics and the residential green environment

Descriptive characteristics of the study population, STEPS Study from Finland^31^, are shown in Table 1. The mean age of the mothers (n = 795) providing milk samples was 31.2 (SD 4.4) years and most mothers (60.8%) had no previous children. Almost 90% of the mothers gave birth to the child via vaginal delivery, the sex-ratio of the children was close to equal and a majority of the children were born at full-term (96.6%). The mean maternal pre-pregnancy BMI was within the limits of normal weight (BMI < 25), only a small minority of mothers smoked before or during pregnancy (7.7%), and most mothers had no chronic diseases or pregnancy-related conditions. A vast majority of the mothers were married or cohabiting and had completed tertiary education. The milk samples were collected on average 2.64 (SD 0.41) months post-partum. The sample collection was equally distributed throughout the seasons. Almost 90% of the mothers exhibited high abundance of the HMO 2′FL in their milk samples and, consequently, were secretors. Furthermore, there were only small differences across child or maternal characteristics and residential green environment variables (Table 1). However, some differences were detected and mothers with previous children, basic education or full-time mothers lived in residential areas with higher NDVI and NI. With regard to milk sample characteristics, secretor mothers were more likely to live in areas with higher NDVI and NI (Table 1).

**Table 1:** Descriptive characteristics of the STEPS Study mothers who provided milk samples, and their infants in relation to residential green environment properties (n = 795) (750 m × 750 m grids).

Variable	n (%)/Mean (SD)	Mean (SD)		
	All	NDVI	VCDI	NI
**Child characteristics**				
Sex				
Boy	428 (53.8)	0.56 (0.12)	0.42 (0.13)	3.03 (0.85)
Girl	367 (46.2)	0.56 (0.13)	0.41 (0.13)	3.02 (0.84)
Birth weight (kg)	3.54 (0.50)	–	–	–
Duration of pregnancy (weeks)	39.94 (1.47)	–	–	–
Twin				
No	787 (99.0)	0.56 (0.13)	0.42 (0.13)	3.02 (0.84)
Yes	8 (1.0)	0.59 (0.15)	0.38 (0.05)	3.15 (1.11)
**Maternal characteristics**				
Age	31.16 (4.36)	–	–*	–
Previous children				
No	483 (60.8)	0.54 (0.14)*	0.41 (0.13)	2.95 (0.87)*
Yes (1–5)	312 (39.3)	0.59 (0.10)	0.43 (0.12)	3.14 (0.79)
Birth mode				
Vaginal	698 (87.8)	0.56 (0.13)	0.42 (0.13)	3.00 (0.84)
Cesarean	97 (12.2)	0.57 (0.12)	0.42 (0.11)	3.16 (0.86)
Pre-pregnancy BMI	24.09 (4.52)	–*	–	–*
Marital status				
Married	488 (62.5)	0.56 (0.12)	0.42 (0.12)	3.05 (0.85)
Cohabiting	267 (34.2)	0.55 (0.13)	0.41 (0.13)	2.98 (0.84)
Single/widow/divorced	26 (3.3)	0.55 (0.14)	0.41 (0.15)	2.95 (0.89)
Education				
Basic	242 (31.0)	0.57 (0.12)*	0.42 (0.12)	3.12 (0.84)*
Advanced	539 (69.0)	0.55 (0.13)	0.42 (0.13)	2.98 (0.85)
Occupation				
Higher-grade non-manual	250 (31.5)	0.56 (0.13)*	0.41 (0.13)	3.03 (0.86)*
Lower-grade non-manual	182 (22.9)	0.57 (0.12)	0.43 (0.12)	3.09 (0.86)
Manual	139 (17.5)	0.58 (0.12)	0.43 (0.12)	3.15 (0.81)
Student	100 (12.6)	0.52 (0.13)	0.40 (0.14)	2.79 (0.80)
Full-time mother	18 (2.3)	0.61 (0.12)	0.42 (0.11)	3.22 (0.95)
Missing	106 (13.3)	0.54 (0.13)	0.42 (0.13)	2.93 (0.83)
Family net income/month				
< 3000 €	409 (53.0)	0.56 (0.12)	0.42 (0.13)	3.00 (0.83)
≥ 3000 €	363 (47.0)	0.56 (0.13)	0.41 (0.12)	3.05 (0.88)
Any diseases^1^				
No	646 (81.3)	0.56 (0.13)	0.42 (0.13)	3.02 (0.84)
Yes	149 (18.7)	0.56 (0.12)	0.42 (0.11)	3.03 (0.86)
Smoking before/during pregnancy				
No	734 (92.3)	0.56 (0.13)	0.42 (0.13)	3.03 (0.85)
Yes	61 (7.7)	0.56 (0.11)	0.43 (0.12)	3.00 (0.83)
Diet during pregnancy^2^				
Healthy	308 (69.8)	0.56 (0.12)	0.42 (0.12)	3.03 (0.82)
Unhealthy	133 (30.2)	0.57 (0.13)	0.42 (0.12)	3.05 (0.84)
**Milk sample**				
Secretor status				
Nonsecretor	102 (12.8)	0.53 (0.14)*	0.41 (0.13)	2.85 (0.80)*
Secretor	693 (87.2)	0.56 (0.12)	0.42 (0.13)	3.05 (0.85)
Lactation time postpartum (months)	2.64 (0.41)	–	–	–
**Lactation status**				
Exclusive breastfeeding	370 (46.5)	0.56 (0.13)	0.41 (0.13)	3.00 (0.80)
Partial breastfeeding	277 (34.8)	0.56 (0.12)	0.43 (0.12)	3.06 (0.86)
Unknown	148 (18.6)	0.55 (0.13)	0.40 (0.13)	3.02 (0.93)
**Living environment**				
Season				
Summer (March–Sep)	446 (56.1)	0.56 (0.13)	0.42 (0.12)	3.02 (0.87)
Winter (Oct–Feb)	349 (43.9)	0.56 (0.12)	0.42 (0.13)	3.02 (0.82)

Sample sizes and percentages are given for categorical variables and means with standard deviations for continuous variables. Asterisks show significant associations between residential green environment variable and child/maternal or study characteristic (p < 0.05).

NDVI, Normalized Difference Vegetation Index, i.e. greenness; VCDI, Vegetation Cover Diversity; NI, Naturalness Index.

^1^Chronic or gestational-related conditions during pregnancy.

^2^Based on Index of Diet Quality (IDQ)^62^.

### HMO composition and associations with background factors

The total concentration of all HMOs in the milk samples varied from 8103 to 20,512 nmol/ml (median (Q1, Q3): 16,183 (15,276, 17,068)). The concentrations for each separate HMO component in secretor vs. non-secretor mothers in the cohort have been already reported elsewhere^40^ as well as comparison of mothers and infants from this HMO subgroup to the original STEPS Study cohort^40^. HMO diversity ranged between 1.72 and 9.66 (mean (SD): 5.15 (1.53)). Several background factors were associated with HMO diversity, sum of HMOs, HMO-bound sialic acid, HMO-bound fucose and the concentrations of the 19 individual HMOs (Supplementary Tables 1a, b). HMO diversity and concentrations of HMO components were highly variable between secretor status, lactation time post-partum (months), lactation status (exclusive/partial/unknown breastfeeding), child sex, number of previous births, marital status, smoking and pre-pregnancy BMI. Additionally, the milk samples collected during summer time, from mothers with only basic education or history of disease, and children with lower birth weight had higher HMO concentrations. Maternal diet recorded during pregnancy, income and birth mode were not associated with any HMO component.

### HMO composition and residential green environments

The mean values of the residential green environment variables in the 750 m × 750 m grids around the homes of the families were: NDVI, 0.56 (SD: 0.13) (range: 0.14–0.78); VCDI, 0.42 (SD: 0.13) (range: 0.02–0.67) and NI, 3.02 (SD: 0.85) (range: 1.11–5.67). Although the variables measure slightly different properties in the residential environments they were moderately correlated (Suppl. Table 2). Adjusted models (N = 772) (including secretor status, season, lactation time, lactation status, child sex, child birth weight, birth mode, duration of pregnancy, number of previous births, marital status, occupation, education, smoking, pre-pregnancy BMI, diseases) showed several associations between residential green environment properties and HMO composition (significant associations with the main term, interaction term with secretor status or curvilinear term, standardized estimates in Table 2 and Supplementary Tables 3a–c, original log estimates in Suppl. Tables 4 and 5a–c). Models adjusting only for secretor status showed very similar results (Suppl. Tables 6a, b). Additionally, adjusted models with smaller grid size in the residential areas, 250 m × 250 m, showed similar, but weaker associations with HMO components (Suppl. Tables 7a, b, 8a–c).

**Table 2:** Adjusted standardized estimates (95% confidence interval) in HMO diversity, individual HMO concentrations and HMO structural groups (nmol/mL) per one SD increase in residential green environment (exposure) variables.

N = 772	NDVI, normalized difference vegetation index		VCDI, vegetation cover diversity		NI, naturalness index	
	Est. (95% CL)	p-value	Est. (95% CL)	p-value	Est. (95% CL)	p-value
Diversity	0.044 (− 0.027, 0.117)	0.23	0.085 (0.015, 0.155)	**0.017**	0.101 (0.030, 0.171)	**0.005**
Sum of HMOs	− 0.002 (− 0.029, 0.024)	0.87	− 0.019 (− 0.049, 0.063)	0.13	− 0.019 (− 0.044, 0.005)	0.14
HMO-bound sialic acid	0.039 (− 0.025, 0.099)	0.23	0.014 (− 0.046, 0.074)	0.66	0.060 (− 0.004, 0.120)	0.053
HMO-bound fucose	0.001 (− 0.029, 0.031)	0.92	− 0.005 (− 0.034, 0.024)	0.75	− 0.005 (− 0.034, 0.024)	0.76
2′FL	− 0.021 (− 0.041, − 0.001)	0.036*	− 0.026 (− 0.045, − 0.007)	0.010*	− 0.025 (− 0.044, − 0.005)	**0.015**
3FL	− 0.001 (− 0.054, 0.053)	0.97	− 0.018 (− 0.070, 0.035)	0.51	− 0.01 (− 0.062, 0.043)	0.72^**C**^
LNnT	0.065 (− 0.007, 0.137)	0.079	0.065 (− 0.007, 0.134)	0.077	0.062 (− 0.010, 0.132)	0.087
3′SL	0.018 (− 0.053, 0.089)	0.62	− 0.034 (− 0.101, 0.036)	0.35	− 0.002 (− 0.070, 0.070)	0.99^**C**^
DFLac	0.022 (− 0.007, 0.051)	0.14	0.032 (0.004, 0.061)	0.026*	0.024 (− 0.005, 0.053)	0.10
6'SL	0.005 (− 0.061, 0.071)	0.87	− 0.009 (− 0.073, 0.057)	0.81*	0.036 (− 0.029, 0.102)	0.28
LNT	0.014 (− 0.055, 0.085)	0.69	0.065 (0.002, 0.134)	0.060	0.037 (− 0.032, 0.107)	0.29
LNFP I	− 0.004 (− 0.045, 0.035)	0.82	− 0.002 (− 0.041, 0.038)	0.94	− 0.016 (− 0.055, 0.024)	0.43*^C^
LNFP II	0.045 (− 0.017, 0.109)	0.15	0.036 (− 0.026, 0.098)	0.24	0.045 (− 0.017, 0.106)	0.16
LNFP III	− 0.024 (− 0.088, 0.041)	0.48	− 0.006 (− 0.070, 0.057)	0.85	− 0.031 (− 0.096, 0.033)	0.35
LSTb	0.054 (− 0.013, 0.041)	0.12	0.106 (0.040, 0.171)	**0.002**	0.096 (0.031, 0.164)	**0.005**
LSTc	0.016 (− 0.055, 0.079)	0.70	− 0.012 (− 0.081, 0.055)	0.72*	− 0.016 (− 0.085, 0.053)	0.65*
DFLNT	0.070 (0.002, 0.143)	**0.042**	0.057 (− 0.008, 0.124)	0.089	0.087 (0.020, 0.154)	**0.011**
LNH	− 0.014 (− 0.087, 0.058)	0.70	0.022 (− 0.049, 0.093)	0.54	− 0.025 (− 0.096, 0.046)	0.49
DSLNT	0.057 (− 0.016, 0.127)	0.12	0.08 (0.012, 0.150)	**0.024**	0.082 (0.012, 0.152)	**0.023**
FLNH	− 0.025 (− 0.093, 0.043)	0.47*	0.016 (− 0.052, 0.083)	0.65	− 0.004 (− 0.071, 0.063)	0.92*
DFLNH	0.018 (− 0.043, 0.078)	0.56	0.035 (− 0.024, 0.093)	0.25	0.003 (− 0.057, 0.063)	0.91^**C**^
FDSLNH	0.017 (− 0.047, 0.081)	0.59	0.039 (− 0.023, 0.102)	0.22	0.003 (− 0.061, 0.066)	0.94
DSLNH	− 0.019 (− 0.089, 0.053)	0.60	− 0.055 (− 0.123, 0.014)	0.12*	− 0.024 (− 0.094, 0.044)	0.49*
Small HMOs	− 0.027 (− 0.061, 0.005)	0.11	− 0.053 (− 0.085, − 0.020)	0.002*	− 0.042 (− 0.075, − 0.010)	**0.012**
Type 1 HMOs	0.029 (− 0.033, 0.091)	0.37	0.057 (− 0.005, 0.117)	0.074	0.033 (− 0.029, 0.095)	0.31^**C**^
Type 2 HMOs	0.060 (− 0.012, 0.133)	0.093	0.060 (− 0.009, 0.130)	0.089	0.052 (− 0.017, 0.121)	0.15
α-1-2-fucosylated HMOs	− 0.018 (− 0.038, 0.002)	0.077	− 0.021 (− 0.041, − 0.001)	**0.033**	− 0.030 (− 0.049, − 0.010)	0.003*
Terminal α-2-6- sialylated HMOs	0.006 (− 0.060, 0.072)	0.86	− 0.014 (− 0.078, 0.050)	0.68*	0.032 (− 0.034, 0.096)	0.35
Internal α-2-6-sialylated HMOs	0.062 (− 0.008, 0.133)	0.079	0.091 (0.023, 0.160)	**0.010**	0.091 (0.021, 0.160)	**0.010**
Terminal α-2-3-sialylated HMOs	0.044 (− 0.026, 0.114)	0.22	0.009 (− 0.058, 0.079)	0.79	0.041 (− 0.029, 0.111)	0.24

Standardization means that both the exposure and outcome have been standardized (log scale in all other outcome variables expect Diversity, which is in the original scale). The exposure variables were measured at the time of child was born (2008–2010) with 750 m × 750 m grid size around the homes of the families. Statistics are given for main effects and significant values are in bold. * Indicates a significant interaction between the exposure variable and secretor status (p < 0.05). ^C^ Indicates a significant curvilinear association with the exposure variable (p < 0.05). Models were adjusted for secretor status, season, lactation time, lactation status, child sex, child birth weight, birth mode, duration of pregnancy, number of previous births, marital status, occupation, education, smoking, pre-pregnancy BMI, diseases.

2′-fucosyllactose (2′FL), 3-fucosyllactose (3FL), lacto-N-neotetraose (LNnT), 3′-sialyllactose (3′SL), difucosyllactose (DFlac), 6′-sialyllactose (6′SL), lacto-N-tetraose (LNT), lacto-Nfucopentaose (LNFP) I, LNFP II, LNFP III, sialyl-LNT (LST) b, LSTc, difucosyllacto-LNT (DFLNT), lacto-N-hexaose (LNH), disialyllacto-N-tetraose (DSLNT), fucosyllacto-Nhexaose (FLNH), difucosyllacto-N-hexaose (DFLNH), fucodisialyllacto-lacto-N-hexaose (FDSLNH) and disialyllacto-N-hexaose (DSLNH). HMO groups: small HMOs (2’FL, 3FL, 3′SL, 6′SL, and DFLac), type 1 HMOs (LNT, LNFP I, LNFP II, LSTb, DSLNT), type 2 HMOs (LNnT, LNFP III, LSTc), α-1-2-fucosylated HMOs (2′FL, LNFP I), terminal α-2-6-sialylated HMOs (6′SL, LSTc), internal α-2-6-sialylated HMOs (DSLNT, LSTb), terminal α-2-3-sialylated HMOs (3′SL, DSLNT).

NDVI was associated with concentrations of three individual HMOs: 2’FL, DFLNT and FLNH (Table 2, Suppl. Table 4, Suppl. Fig. 1a). One SD increase in NDVI was associated with increased DFLNT concentration but decreased 2’FL concentration. The associations were also different in non-secretor and secretor mothers indicated by the significant interaction in 2’FL and FLNH (Suppl. Table 3a and 5a, Suppl. Fig. 1a). NDVI was not associated with any HMO structural groups (Table 2, Suppl. Tables 3a, 4, 5a).

VCDI was associated with HMO diversity and seven individual HMO components: 2′FL, DFLac, 6′SL, LSTb, LSTc, DSLNT, DSLNH (Table 2, Suppl. Table 4, Suppl. Fig. 1b). One SD increase in VCDI was associated with increased HMO diversity. Similarly, one SD increase in VCDI was associated with increased concentrations of DFLac, LSTb and DSLNT, but decreased 2′FL. The associations with VCDI also differed between non-secretor and secretor mothers in 2′FL, DFLac, 6′SL, LSTc and DSLNH (significant interactions with secretor status, Suppl. Tables 3b and 5b, Suppl. Fig. 1b). HMO group analyses showed positive association of VCDI with internal α-2-6-sialylated HMOs and negative associations with small HMOs and α-1-2-fucosylated HMOs (Table 2, Suppl. Table 4). The associations were also different in non-secretor and secretor mothers indicated by significant interactions in small HMOs and terminal α-2-6-sialylated HMOs (Table 2, Suppl. Table 3b, 4, 5b, Suppl. Fig. 1b).

Finally, NI was associated with HMO diversity and 11 individual HMO components, 2′FL, 3FL, 3′SL, LNFP I, LSTb, LSTc, DFLNT, DSLNT, FLNH, DFLNH, DSLNH (Table 2, Suppl. Table 4, Suppl. Fig. 1c). One SD higher NI was associated with increased HMO diversity. One SD increase in NI was also associated with increased concentrations of LSTb, DFLNT and DSLNT but decreased concentration of 2′FL. The associations of NI differed between non-secretor and secretor mothers in LNFPI, LSTc, FLNH and DSLNH (significant interactions with secretor status, Suppl. Tables 3c, 5c, Suppl. Fig. 1c). Curvilinear associations were found between NI and 3′FL, 3′SL, LNFPI and DFLNH (Suppl. Tables 3c, 5c). HMO group analyses showed that one SD increase in NI was associated with higher concentration of internal α-2-6-sialylated HMOs and decreased concentration in small HMOs and α-1-2-fucosylated HMOs (Table 2, Suppl. Table 4, Suppl. Fig. 1c). Different associations in non-secretor and secretor mothers with NI were found in α-1-2-fucosylated HMOs and curvilinear association with NI in type 1 HMOs (Table 2, Suppl. Tables 3c, 4, 5c, Suppl. Fig. 1c).

**Figure 1:**
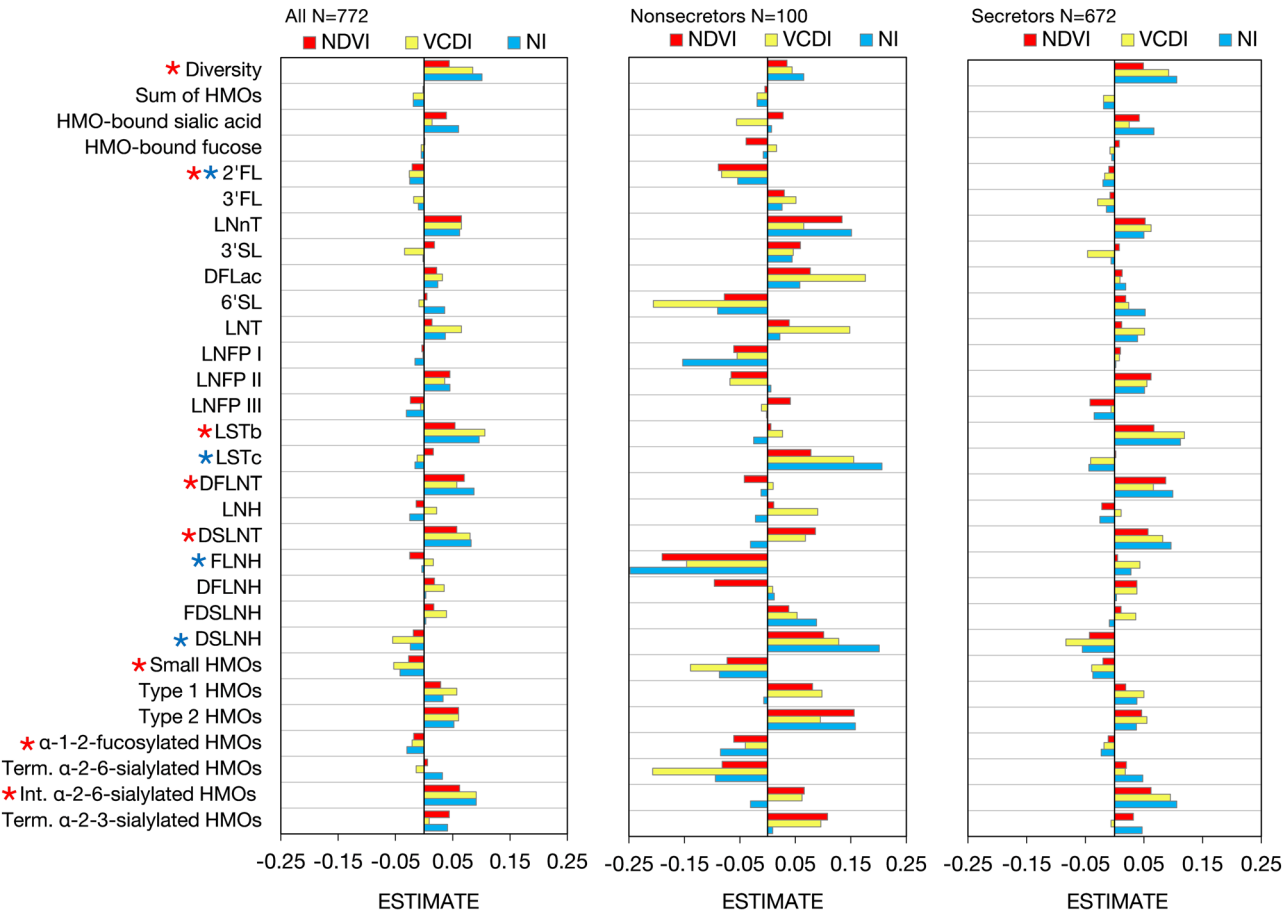
Associations between residential green environment and HMO diversity, individual HMO concentrations and HMO structural groups (750 m × 750 m grids). Figures are plotted separately for all (estimates from main effects, Table 2), and non-secretors and secretors (estimates from interactions, Suppl. Table 3a–c). Size of the bar shows the strength of the association and negative and positive values the direction of the association by standardized estimates. Red stars indicate ≥ 2/3 of the main associations with the HMO component and the three residential green environment variables were significant, and blue stars that ≥ 2/3 of the interactions between the residential green environment variables and secretor status being significant i.e. associations with residential green environment on HMO component concentration differ by secretor status. Term., terminal; Int., internal.

In sum, characteristics of the residential green environments were mostly associated with the same HMO components, measured individually or in structurally defined groups (at least two of the associations (main effects) with the three residential green environment variables were significant in these HMO components): HMO diversity, individual HMOs: 2′FL, LSTb, DFLNT, DSLNT; HMO groups: small HMOs, α-1-2-fucosylated HMOs, internal α-2-6-sialylated HMOs) (Fig. 1). The direction of the association was also mostly the same between all residential green environment variables, estimates becoming often stronger with higher VCDI and/or NI (Fig. 1; higher bars). However, many of the associations with residential green environment variables and HMO components were more evident in non-secretor mothers than secretor mothers, indicated by the significant interactions with secretor status (≥ 2/3 significant interactions with residential green environment variables: 2′FL, LSTc, FLNH, DSLNH) (Fig. 1). Additional models including residential socioeconomic disadvantage as an additional confounder showed similar results (Suppl. Table 9a, b with standardized and original log estimates, respectively).

## Discussion

**Figure 2:**
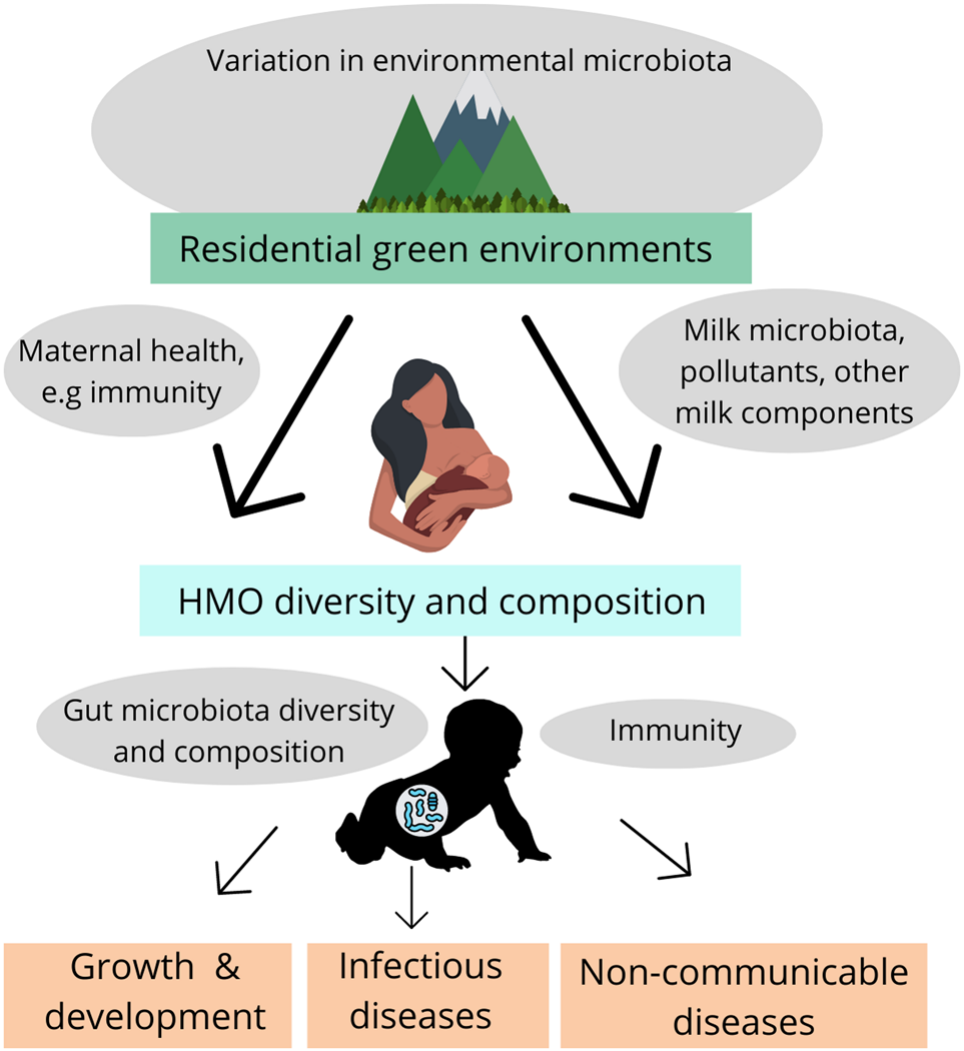
Potential pathways from residential green environment to infant health via variations in HMO composition. The investigated associations in this study are shown with bold arrows. The thin arrows show suggested pathways, grey ovals physiological mechanisms and orange boxes infant health outcomes, which need further studies. Green environments (with varying levels of environmental microbiota)^35,44^ may influence the abundance and composition of microbiota that colonize the maternal skin and respiratory tract leading to changes in maternal immunity^26–28,32,34^ and milk microbiota^42^. Green environment may also influence the concentrations of other components in milk such as pollutants^12^ which interact with HMOs within milk. These lead to changes in HMO diversity and composition. Modifications in HMO composition may further impact on infant gut microbiota composition^46^ and immunity^20^ impacting on risks of several childhood diseases.

In this work, we show associations between residential green environment and HMO composition in a Finnish cohort of breastfeeding women. Previously, it has been found that HMO composition is associated with wide scale environmental variables measured as cities, countries, urban/rural areas or seasons^23–25^. We demonstrate that greater diversity and proportion of green environments in the residential area were associated with greater HMO diversity and changes in the concentrations of several HMO structural groups as well as individual HMOs. Some significant differences were detected between secretor and non-secretor mothers (classified by the high abundance or near-absence of the HMO 2′FL), and many of the associations with the residential green environment were stronger in non-secretor mothers. Our results are suggestive of a potential pathway between residential green environments and HMO composition which may have subsequent effects on child health, but this needs to be investigated in further studies (Fig. 2). Residential green environments with potentially higher microbial diversity could enhance maternal immunity^26–28,41^ and increase the abundance of immunological compounds^32^ including HMOs in maternal milk. High microbial diversity in the environment is also likely to increase milk bacterial diversity^42^ (or associate with pollutants^12^ or fungi^13^) which may further influence HMO composition through interactions within milk^12^. Environment-related variation in HMO composition may lead to further changes in infant gut microbiota composition and risk of several diseases. The suggested links to infant immunity, gut microbiota composition and health are currently speculative as they were not investigated in this study. Our results show an association between residential green environment and HMO composition, which might be part of a previously largely unexplored environmental signaling mechanism via maternal milk.

To our knowledge, only one previous study has examined human milk composition in relation to the properties of the residential green environment (with 250 m buffer zone from mother’s home), showing that human milk fungi were not associated with residential NDVI^13^. Our finding that the strongest associations with HMO diversity and concentrations were observed with VCDI and NI, and weaker associations were observed with NDVI, are in line with this previous study and reflect the fact that NDVI is a generic measure of green vegetation, and its density and vitality, but it does not directly identify vegetation types and plant composition. Additionally, NDVI is only calculated during summer time in Finland whereas the milk samples were collected around the year. However, NDVI captures multiple characters of green space in the residential neighborhood rather than characteristics of environments only specific to summer time. Moreover, exposure to green spaces is relatively high in Finland also in urban areas compared to many other European cities^43^, and only a small percentage of families (5.7%) in our study population lived in low greenness residential areas (NDVI < 0.3). Thus, linking NDVI to specific properties of green environments and HMO composition has its limitations. Some specific green land cover types or their combinations (e.g. forests, grasslands, shrubs, mires) as well as complex vegetation communities within the green environment have been shown to associate with higher levels of microbiota diversity in air and soil^35,44^. As our VCDI measure includes six land cover classes indicating green environments (CORINE) and estimates their diversity (Agriculture, Broad-leave forest, Coniferous forest, Mixed forest, Shrub/grassland, Wetlands), the variable is likely to indicate the complexity of vegetation and potentially microbial diversity in the residential area, although specific studies to establish such relationships have to date not been carried out. Low NI values, in our study, represented mainly impervious asphalt covered residential and industrial areas, while high NI values included non-built areas, e.g., natural bare surfaces, water bodies, wetlands and different types of forests^38^. The associations we observed between NI and HMO composition suggests, that not only green spaces but also other natural features of the residential area, such as the presence of water areas and wetlands, may have an important role in HMO composition.

Our study found that HMO diversity, several HMO groups and individual HMOs were associated with residential green environment, particularly with VCDI and NI. Firstly, HMO diversity, a measure of the abundance and variety of HMOs, increased with increasing VCDI and NI. Secondly, we observed positive associations between internal α-2-6-sialylated HMOs (DSLNT, LSTb) and residential green environment. In this context the concentrations increased with increasing VCDI and NI. Thirdly, we found that small HMOs (2′FL, 3FL, 3′SL, 6′SL, and DFLac) and α-1-2-fucosylated HMOs (2′FL, LNFP I) showed negative associations with VCDI and NI. Although the associations between HMO composition and properties of the residential green environments have to our knowledge not been previously explored, our results are in line with the findings from a study including 11 international cohorts from developed and developing countries^23^ (INSPIRE Study). This study found that the lowest HMO diversities were observed in urban and sub-urban areas of Sweden, USA and Peru and highest HMO diversities in Ghana, Ethiopia, Kenya and Gambia. Our mean diversity value was closer to values observed in other western societies. The INSPIRE study^23^ also found that DSLNT concentrations were higher in rural than urban Gambia, similar to our finding that DSLNT increases with VCDI and NI. Finally, the INSPIRE study found that 2′FL concentration were similarly high compared to our study, around 6000 nmol/ml, in Sweden, USA and Peru and much lower particularly in populations from rural Africa (< 3000 nmol/ml)^23^. Previous studies have also found substantial variation between geographical locations in the concentrations of fucosylated HMOs^45^. It has been suggested that variations in environment, diet and lifestyle all contribute to these observed differences across geographic locations although genetics (and prevalence of secretors) may also explain at least part of these differences in HMOs. However, observations that human milk immune profiles also differ between the same countries, suggest that exposure to environmental factors including microbiota might drive a part of the differences. For instance, milk immune profiles differed vastly between developed and developing countries as well as rural and urban areas in Gambia in the INSPIRE Study (greater immune response plasticity in developing and rural areas), and this was suggested to result from differential exposure to environmental microbiota between these locations^32^.

Our data suggest that the associations with residential green environments may vary by maternal genetics (secretor vs non-secretor). The associations observed in this study were often stronger in the non-secretor mothers, and the directions of some associations were opposite in secretors and non-secretors. Non-secretor mothers are capable of producing only small amounts of 2′FL and LNFP-I but their HMO profile differs from secretor mothers also by the amounts of several other HMO components. In our study, secretor status was significantly linked with 17/19 individual HMO components. The non-secretor phenotype is more prevalent in Africa, Central Asia, Far East and Pacific regions compared to other regions, and this is suggested to be due to the fact that such trait might have provided protection against certain viruses or other pathogens in these areas^21^. The variation in HMO composition among women has in fact been suggested to result from both genetic and environmental variation that respond to different selective pressures^21^. In our study, secretor mothers lived in greener environments compared to non-secretor mothers (50% vs. 43% lived in the greenest areas (NDVI > 0.6)), suggesting the strength of the HMO associations with green environments may also differ with the exposure levels to green environment. Further studies are needed to understand whether the susceptibility to respond to environmental cues depends on the genetics, and exposure levels, and how these associations reflect on infant health.

Accumulating evidence suggests that some of the associations we found between residential green environment and HMO composition may be further connected to beneficial composition of infant fecal microbiota and improved infant health outcomes. Higher HMO diversity with higher VCDI and NI potentially increases gut microbiota diversity as HMOs are linked to gut microbiota composition through acting as prebiotics^19,46^. Gut microbiota diversity has been reported to be greater in microbe rich environments^26,29^, and living areas with diverse vegetation cover (higher diversity of yard shrub species) were negatively associated with dysbiotic shifts in the gut microbiota^47^. Higher gut microbiota diversity in early life has been associated with reduced risk of health concerns such as asthma^48^ and allergic diseases^49^. Furthermore, higher HMO diversity and higher concentrations of HMOs have been connected directly to lower risks of several diseases in infancy (e.g. necrotizing enterocolitis (NEC)^50,51^, bacterial and viral infections^52^ and immune-mediated diseases^20,53^). Moreover, Lagström et al. 2020 found in this same study population that higher HMO diversity but lower concentrations of 2’FL were associated with lower height and weight in early childhood in Finland^40^. These results thus suggest that higher HMO diversity and lower concentrations of 2′FL with higher VCDI and NI may protect from obesity development in children in this western population. In order to bring clarity to these debated topics further studies are needed to investigate the specific links between residential green environments, gut microbiota diversity and infant health outcomes taking into account the variation in HMO composition.

There are some important issues that need to be considered when interpreting the results and implications of the present study. Firstly, although we speculate that the residential green environment may be associated with HMO composition via environmental microbial diversity, there are other routes that could have led to these associations. Residential green environment may modify the HMO composition by e.g. increasing physical activity of mothers and relieving their mental stress^30,54^. We did not have information from mother’s physical activity levels at the time of milk collection. However, we tested associations between HMOs and residential socioeconomic disadvantage, which has been connected to increased risk of mental disorders^55^, and found no clear evidence of a link between the two. Secondly, the effect sizes between residential green environment and HMO composition were relatively small, not allowing corrections for multiple testing. However, it should be noted that the use of corrections is debated^56^. Thirdly, we only had one milk sample from each mother so we could not investigate within mother variation in HMO composition in relation to changing residential location, which would have made the study stronger. Finally, less than 50% of mothers were known to exclusively breastfeed their children at the time of milk collection, which may have impacted the HMO composition. However, we have adjusted the analyses with lactation status, as well as many other confounding factors known to be associated with HMO composition and we therefore believe these variables are unlikely to confound the results between residential green environment and HMO composition. Furthermore, our study benefits from detailed information on maternal and child characteristics, objectively determined accurate place of residence linked to specific measures on the surrounding green environment and similar findings from two different buffer zones around the homes of mothers and their children (250 m × 250 m and 750 m × 750 m grid sizes). The findings were also similar in models with only secretor status as an adjusted variable, suggesting that the results from this study are robust. Furthermore, the participating families, whose children were born in 2008–2010, lived in a geographically concentrated area in Southwest Finland. Thus, the data is explicit in space and time, increasing the validity of the findings in this homogenous study population.

In conclusion, our results demonstrate that HMO diversity increased and the concentrations of individual HMOs and HMO groups changed with increased exposure to residential green environments. The findings are important for future studies on infant health as HMOs have a crucial role in the development of the neonatal immune system. The findings emphasize the need to understand the biological pathways that lead to the development of health and disease via both direct and indirect contact with the physical environment, including via exposure to diverse milk compositional profiles across different environments. Our results imply that increased everyday contacts with nature might be beneficial for breastfeeding mothers by increasing HMO diversity. These findings also point to the need of new nature-based clinical trials. Overall further studies with a focus on the properties of residential green environment, but also taking into account house and indoor characteristics, and HMO composition in maternal milk, are needed. These should be conducted across different populations and climates, in order to determine which characteristics of the residential environment and which exposure times provide the greatest public-health benefits and contribute to a balanced infant immunity and to improved health outcomes.

## Methods

### Study population

The study is based on data from mothers and children participating in a longitudinal Southwest Finland cohort, Steps to Healthy development of Children (the STEPS Study) (described in detail in Lagström et al.^31^). The STEPS study is an ongoing population-based and multidisciplinary study that investigates children's physical, psychological and social development, starting from pregnancy and continuing until adolescence. All Finnish- and Swedish-speaking mothers delivering a child between 1 January, 2008 and 31 March, 2010 in the Hospital District of Southwest Finland formed the cohort population (in total, 9811 mothers and their 9936 children). Altogether, 1797 mothers with 1805 neonates volunteered as participants for the intensive follow-up group of the STEPS Study. Mothers were recruited by midwives either during the first trimester of pregnancy while visiting maternity health care clinics, or after delivery on the maternity wards of Turku University Hospital or Salo Regional Hospital, or by a letter mailed to the mothers. The participating mothers differ slightly from the whole cohort population in some background characteristics (being older, with first-born child and higher socioeconomic status)^31^. The ethics committee of the Hospital District of Southwest Finland has approved the STEPS Study (2/2007) and all methods were performed in accordance with relevant guidelines and regulations. Written informed consent was obtained from all the participants and, for children, from one parent for study participation. Subjects have been and are free to withdraw from the study at any time without any specific reason. The STEPS Study have the appropriate government authorization to the use of the National birth register (THL/974/5.05.00/2017).

### Breastmilk collection and HMO analysis

Mothers from the STEPS Study were asked to collect breastmilk samples when the infant was approximately 3 months old. In total, 812 of the 1797 mothers (45%) provided a breastmilk sample. There were only slight differences in maternal and child characteristics between the participants providing breastmilk samples and the total STEPS Study cohort^40^. Altogether, 795 breastmilk samples were included in this study (excluding the duplicate observations and the 2^nd^ born twins, samples with technical unclarity or insufficient sample quantity, one breastmilk sample collected notably later than the other samples, at infant age of 14.5 months (range for the other breastmilk samples: 0.6–6.07 months), one sample with missing information on the date of collection and six mothers missing data on residential green environment) (Supplementary Fig. 2). Mothers received written instructions for the collection of breastmilk samples: samples were collected by manual expression in the morning from one single breast, first milking a few drops to waste before collecting the actual sample (~ 10 ml) into a plastic container (pre-feed sample). The samples were stored in the fridge and the mothers brought the samples to the research center or the samples were collected from their homes on the day of sampling. All samples were frozen and stored at − 70 °C until analysis.

High Performance Liquid Chromatography (HPLC) was used to identify HMOs in breastmilk as previously described^40,57,58^ at the University of California, San Diego (methods described in detail in Berger et al.^58^). Milk samples were spiked with raffinose (a non-HMO carbohydrate) as an internal standard to allow absolute quantification. HMOs were extracted by high-throughput solid-phase extraction, fluorescently labelled, and measured using HPLC with fluorescent detection (HPLC-FLD)^58^. Absolute concentrations for the 19 HMOs were calculated based on standard retention times and corrected for internal standard recovery. Quantified HMOs included: 2′-fucosyllactose (2′FL), 3-fucosyllactose (3FL), lacto-N-neotetraose (LNnT), 3′-sialyllactose (3′SL), difucosyllactose (DFlac), 6′-sialyllactose (6′SL), lacto-N-tetraose (LNT), lacto-Nfucopentaose (LNFP) I, LNFP II, LNFP III, sialyl-LNT (LST) b, LSTc, difucosyllacto-LNT (DFLNT), lacto-N-hexaose (LNH), disialyllacto-N-tetraose (DSLNT), fucosyllacto-Nhexaose (FLNH), difucosyllacto-N-hexaose (DFLNH), fucodisialyllacto-lacto-N-hexaose (FDSLNH) and disialyllacto-N-hexaose (DSLNH). HMOs were also summed up to seven groups based on structural features: small HMOs (2′FL, 3FL, 3′SL, 6′SL, and DFLac), type 1 HMOs (LNT, LNFP I, LNFP II, LSTb, DSLNT), type 2 HMOs (LNnT, LNFP III, LSTc), α-1-2-fucosylated HMOs (2’FL, LNFP I), terminal α-2-6-sialylated HMOs (6′SL, LSTc), internal α-2-6-sialylated HMOs (DSLNT, LSTb), terminal α-2-3-sialylated HMOs (3′SL, DSLNT). The total concentration of HMOs was calculated as the sum of the 19 oligosaccharides. HMO-bound fucose and HMO-bound sialic acid were calculated on a molar basis. The proportion of each HMO comprising the total HMO concentration was also calculated. HMO Simpson’s diversity was calculated as Simpson’s Reciprocal Index 1/D, which is the reciprocal sum of the square of the relative abundance of each of the measured 19 HMOs^57,59^. The higher the diversity value, the more heterogenous is the HMO composition in the sample.

### Properties of the residential green environment

The selected residential green environment variables measure the properties of the green environments surrounding the homes of the participants and do not include any measures of the house characteristics, indoor environment or the actual use of green spaces by the participants. The residential green environment variables were selected due to their previously observed associations with residential microbiota and health^33–35^. The variables of the residential green environments were derived from multispectral satellite images series, with a 30 m × 30 m of spatial resolution (Landsat TM 5, National Aeronautics and Space Administration—NASA) and land cover data (CORINE). We used Landsat TM images obtained over the summertime (June–August, greenest months in Finland), to minimize the seasonal variation of living vegetation and cloud cover as well as to better identify vegetation areas and maximise the contrast in our estimated exposure. In each selected Landsat TM 5 images, the cloud was masked out, and the Normalized Difference Vegetation Index (NDVI)^36^ was calculated. The final NDVI map was the mean of NDVI images collected over three consecutive years (2008–2010), to make an NDVI map with non-missing values due to cloud cover for the study area. NDVI map measures the vegetation cover, vitality and density. The NDVI can get values ranging from − 1 to 1 where values below zero represent water surfaces, values close to zero indicate areas with low intensity of living vegetation and values close to one indicate high abundance of living vegetation. For the analyses, areas covered by water were removed and the value ranged from 0 to 1, to prevent negative values for underestimating the greenness values of the residential area like in some prior studies^60^. We assumed that summertime NDVI identified the green space and vegetation density well, but greenness intensity might vary seasonally.

Second, we used calculated indicators related to the diversity and naturalness of the land cover from CORINE Land Cover data sets of the year 2012^61^. The 12 land cover types include: (1) Residential area, (2) Industrial/commercial area, (3) Transport network, (4) Sport/leisure, (5) Agriculture, (6) Broad-leave forest, (7) Coniferous forest, (8) Mixed forest, (9) Shrub/grassland, (10) Bare surface, (11) Wetland, and (12) Water bodies. From this information, we calculated two vegetation cover indexes. The Vegetation Cover Diversity Index (Simpson's Diversity Index of Vegetation Cover, VCDI)^37^, only includes vegetation classes from CORINE land cover types (categories 5–9 and 11). VCDI approaches 1 as the number of different vegetation classes increases and the proportional distribution of area among the land use classes becomes more equitable. Furthermore, because we were particularly interested in the natural vegetation cover in the residential area, we calculated the area-weighted Naturalness Index (NI)^38^. This is an integrated indicator used to measure the human impact and degree of all human interventions on ecological components. The index is based on CORINE Land Cover data but reclassified to 15 classes. Residential areas have been divided to two classes: Continuous residential area (High density buildings) and Discontinuous residential area (Low density, mostly individual houses area). Agricultural area has also been divided to two classes: Agricultural area (Cropland) and Pasture as well as class 9 (Shrub/grassland) has been separated to Woodland and Natural grassland. Assignment of CORINE Land Cover classes to degrees of naturalness has been made based on Walz and Stein 2014^38^. The area-weighted NI range from 1 to 7, where low values represent low human impact (≤ 3 = Natural), medium values moderate human impact (4–5 = Semi-natural) and high values strong human impact (6–7 = Non-Natural). To ease the interpretation of results and to correspond to the same direction than the other residential green environment variables, we have reverse-scaled the NI values, so that higher values illustrate more natural residential areas.

### Background factors

As genetics is strongly linked to HMO composition, maternal secretor status was determined by high abundance (secretor) or near absence (non-secretor) of the HMO 2’FL in the breastmilk samples. Mothers with active secretor (Se) genes and FUT2 enzyme produce high amounts of α-1-2-fucosylated HMOs such as 2′-fucosyllactose (2′FL), whereas in the breastmilk of non-secretor mothers these HMOs are almost absent. Beyond genetics, other maternal and infant characteristics may influence HMO composition. So far, several associations have been reported, including lactation stage, maternal pre-pregnancy BMI, maternal age, parity, maternal diet, mode of delivery, infant gestational age and infant sex^22,40^. Information on the potential confounding factors, child sex, birth weight, maternal age at birth, number of previous births, marital status, maternal occupational status, smoking during pregnancy (before and during pregnancy), maternal pre-pregnancy BMI, mode of delivery, duration of pregnancy and maternal diseases [including both maternal disorders predominantly related to pregnancy such as pre-eclampsia and gestational diabetes and chronic diseases (diseases of the nervous, circulatory, respiratory, digestive, musculoskeletal and genitourinary systems, cancer and mental and behavioral disorders, according to ICD-10 codes, i.e. WHO International Classification of Diseases Tenth Revision)], were obtained from Medical Birth Registers. Self-administered questionnaires upon recruitment provided information on family net income and maternal education level. Those who had no professional training or a maximum of an intermediate level of vocational training (secondary education) were classified as “basic”. Those who had studied at a University of Applied Sciences or higher (tertiary education) were classified as “advanced”. The advanced level included any academic degree (bachelor’s, master’s, licentiate or doctoral degree). Maternal diet quality was assessed in late pregnancy using the Index of Diet Quality (IDQ^62^) which measures adherence to health promoting diet and nutrition recommendations. The IDQ score was used in its original form by setting the statistically defined cut-off value at 10, with scores below 10 points indicating unhealthy diets and non-adherence and scores of 10–15 points indicating a health-promoting diet and adherence dietary guidelines. Lactation time postpartum (child age) and season were received from the recorded breastmilk collection dates. Lactation status (exclusive/partial/unknown breastfeeding) at the time of breastmilk collection were gathered from follow-up diaries. From partially breastfeeding mothers (n = 277) 253 had started formula feeding and 28 solids at the time of milk collection. Last, a summary z score representing socio-economic disadvantage in the residential area was obtained from Statistics Finland grid database for the year 2009 and is based on the proportion of adults with low level of education, the unemployment rate, and proportion of people living in rented housing at each participant’s residential area^55^.

### Statistical analyses

To harmonize the residential green environment variables we calculated the mean values for NDVI, VCDI and NI in 750 × 750 m squares (and 250 × 250 m) around participant homes in a Geographical Information System (QGIS, www.qgis.org). The same grid sizes were used to calculate residential socioeconomic disadvantage in the residential area^55^ at the time of child birth. The geographical coordinates (latitude/longitude) of the cohort participants’ home address (795 mothers) were obtained from the Population Register Centre at the time of their child birth.

The background characteristics of the mothers and children are given as means and standard deviations (SD) for continuous variables and percentages for categorical variables. Due to non-normal distribution, natural logarithmic transformation was performed for all HMO variables (19 individual components, sum of HMOs, HMO-bound sialic acid, HMO-bound fucose and HMO groups (all in nmol/mL)) except for HMO diversity. Associations between each background factor and HMO diversity and 19 individual HMO components were analysed with univariate generalized linear models to identify factors independently associated with HMO composition. All factors demonstrating a significant association (p < 0.05) with any HMO component were included as potential confounders in the subsequent models of residential green environment and HMO composition. In addition, we selected a priori the background factors observed in previous studies to associate with HMO (secretor status, lactation time and status (exclusive/partial/unknown breastfeeding), infant sex, birth mode, duration of pregnancy, maternal age at birth, maternal pre-pregnancy BMI and number of previous births)^22,24^. Before analyses, to allow for a consistent comparison of regression coefficients, all continuous variables of the residential green environment (NDVI, VCDI, NI) were standardized by subtracting the mean and dividing by the standard deviation. In addition, all associations with HMO composition are presented as standardized beta coefficients with 95% confidence intervals per 1 SD increase in residential green environment variables. For standardized beta coefficients also the outcome (HMO composition) variables have been standardized to get betas comparable between the models (presented in log scale in all other outcome variables expect Diversity, which is in the original scale). The multivariable generalized linear models adjusting for all the identified confounding background factors were conducted separately for each residential green environment variable. The analyses also tested for interactions between secretor status and residential green environment variable, to find out whether the green environment is associated differently with HMO composition in secretor vs. non-secretor mothers. In addition, curvilinear associations between the residential green environment variables and HMO composition were tested to investigate any nonlinear associations. Last, as green environment may differ between socio-economically different neighborhoods and thus the results could reflect socio-economic differences between residential areas, we conducted additional analyses for all residential green environment models adjusting for the residential socio-economic disadvantage. The level of significance was set at p value < 0.05. All analyses were conducted using the SAS 9.4 Statistical Package (SAS Institute Inc., Cary, North Carolina).

## Supplementary Information

Supplementary Information.

## Data Availability

The dataset supporting the conclusions of this article can be made available upon request from the corresponding author after approval is obtained from the STEPS Study Executive Committee.
