# Supplementary material for: Residential green environments are associated with human milk oligosaccharide diversity and composition
Source: Sci Rep. 2023 Jan 5;13:216. doi: 10.1038/s41598-022-27317-1 (PMC9816313; doi:10.1038/s41598-022-27317-1)

Supplementary Information

Residential green environments are associated with human milk oligosaccharide diversity and composition

Mirkka Lahdenperä, Laura Galante, Carlos Gonzales-Inca, Jussi Vahtera, Jaana Pentti, Samuli Rautava, Niina Käyhkö, Chloe Yonemitsu, Julia Gupta, Lars Bode, Hanna Lagström

Supplementary table 1a. Significances of the associations between background variables and HMO diversity and individual HMO concentrations (nmol/mL). *p<0.05; **p<0.0001.

Maternal/child characteristics	HMO diversity	Sum of HMOs	HMO-bound sialic	HMO-bound fucose	2'FL	3FL	LNnT	3'SL	DFLac	6'SL	LNT	LNFP I	LNFP II	LNFP III	LSTb	LSTc	DFLNT	LNH	DSLNT	FLNH	DFLNH	FDSLNH	DSLNH
Secretor status, n=801[1]	*	**	**	**	**	**	-	**	**	**	**	**	**	**	**	**	**	-	*	**	**	**	*
Season, n=800	*	-	-	-	-	-	-	-	*	*	-	-	-	-	-	-	-	-	-	*	-	-	*
Lactation time, n=800[1]	-	-	-	-	-	*	-	**	-	-	**	-	*	-	*	**	-	-	-	**	*	-	**
Lactation status, n=801[1]	-	-	*	-	-	-	-	-	-	-	-	-	-	-	*	-	-	*	*	*	-	*	-
Child sex, n=801[1]	*	-	-	-	-	*	*	-	-	-	-	-	-	-	-	-	-	-	-	-	-	-	-
Child birth weight, n=800	*	-	-	-	-	-	-	-	-	-	-	-	*	-	-	-	*	-	*	-	-	-	-
Birth mode, n=800[1]	-	-	-	-	-	-	-	-	-	-	-	-	-	-	-	-	-	-	-	-	-	-	-
Duration of pregnancy, n=800[1]	-	-	-	-	-	-	-	-	-	-	-	-	*	-	-	-	-	-	-	-	-	-	-
Maternal age, n=801[1]	-	-	-	-	-	-	-	-	-	-	-	-	-	-	-	-	-	-	*	-	-	-	-
Previous births, n=801[1]	-	-	*	-	-	-	*	*	-	-	-	-	-	-	-	-	-	-	-	-	-	-	*
Marital status, n=787	-	-	-	-	-	-	-	-	-	-	-	-	-	-	*	-	-	-	*	-	-	*	-
Occupation, n=800	*	-	-	-	-	-	-	-	-	-	-	-	-	-	-	-	-	-	-	-	-	-	-
Education, n=787	-	-	*	-	-	-	-	-	-	-	-	-	-	-	*	*	-	-	*	-	-	-	-
Income, n=778	-	-	-	-	-	-	-	-	-	-	-	-	-	-	-	-	-	-	-	-	-	-	-
Smoking, n=800	*	-	-	-	-	-	-	-	-	-	-	-	-	-	*	-	-	-	*	-	-	*	-
Pre-pregnancy BMI, n=796[1]	-	-	-	-	-	-	-	-	-	-	-	-	-	-	-	*	*	-	-	-	-	-	-
Maternal diet, n=445	-	-	-	-	-	-	-	-	-	-	-	-	-	-	-	-	-	-	-	-	-	-	-
Maternal diseases, n=801	*	-	*	-	-	-	-	-	-	-	-	-	-	-	-	-	-	-	-	-	-	-	-
Socio-economic disadvantage, n=705[2]	-	-	-	-	-	-	-	*	-	-	-	-	-	-	-	-	-	-	-	-	-	-	-

[1]Selected a priori to the HMO models

[2]750 m x 750 m grid size in the residential area, only included in additional analyses

Supplementary table 1b. Heatmap of the statistically significant associations ($p<0.05$, see Table 1a) between background variables and individual HMO concentrations (nmol/mL) (not standardized estimates). Log scale in all other outcome variables expect Diversity, which is in the original scale. Color and shading reflect direction and strength of associations (estimates). Blue= negative; red=positive; white=no association.

Maternal and child characteristics	HMO diversity	Sum of HMOs	HMO-bound sialic acid	HMO-bound fucose	2'FL	3FL	LNnT	3'SL	DFLac	6'SL	LNT	LNFP I	LNFP II	LNFP III	LSTb	LSTc	DFLNT	LNH	DSLNT	FLNH	DFLNH	FDSLNH	DSLNH
Secretor status (secretor), n=801[1]	-0.39	0.58	-0.42	1.04	4.79	1.21	0	0.26	4.42	-0.70	0.31	2.97	-0.68	-0.71	-0.48	0.28	0.81	0	-0.13	0.70	1.08	-0.90	-0.24
Season (summer), n=800	0.25	0	0	0	0	0	0	0	0.24	0.08	0	0	0	0	0	0	0	0	0	0.11	0	0	-0.10
Lactation time, n=800[1]	0	0	0	0	0	0.18	0	0.17	0	0	-0.18	0	0.15	0	0.09	-0.35	0	0	0	-0.26	-0.13	0	-0.35
Lactation status (exclusive)[3], n=801[1]	0	0	-0	0	0	0	0	0	0	0	0	0	0	0	-0.10	0	0	0.16	-0.09	0.11	0	0.12	0
Child sex (girl), n=801[1]	0.23	0	0	0	0	-0.11	0.08	0	0	0	0	0	0	0	0	0	0	0	0	0	0	0	0
Child birth weight, n=800	-0.24	0	0	0	0	0	0	0	0	0	0	0	-0.07	0	0	0	-0.15	0	-0.09	0	0	0	0
Birth mode (vaginal), n=800[1]	0	0	0	0	0	0	0	0	0	0	0	0	0	0	0	0	0	0	0	0	0	0	0
Duration of pregnancy, n=800[1]	0	0	0	0	0	0	0	0	0	0	0	0	-0	0	0	0	0	0	0	0	0	0	0
Maternal age, n=801[1]	0	0	0	0	0	0	0	0	0	0	0	0	0	0	0	0	0	0	-0	0	0	0	0
Previous births (none), n=801[1]	0	0	0.04	0	0	0	-0.09	0.09	0	0	0	0	0	0	0	0	0	0	0	0	0	0	0.12
Marital status (living alone), n=787	0	0	0	0	0	0	0	0	0	0	0	0	0	0	0.25	0	0	0	0.31	0	0	-0.33	0
Occupation (full-time mother), n=800	0.66	0	0	0	0	0	0	0	0	0	0	0	0	0	0	0	0	0	0	0	0	0	0
Education (advanced), n=787	0	0	-0.06	0	0	0	0	0	0	0	0	0	0	0	-0.09	-0.09	0	0	-0.09	0	0	0	0
Income (<3000€), n=778	0	0	0	0	0	0	0	0	0	0	0	0	0	0	0	0	0	0	0	0	0	0	0
Smoking (no), n=800	-0.49	0	0	0	0	0	0	0	0	0	0	0	0	0	-0.20	0	0	0	-0.18	0	0	0.23	0
Pre-pregnancy BMI, n=796[1]	0	0	0	0	0	0	0	0	0	0	0	0	0	0	0	0.01	-0	0	0	0	0	0	0
Maternal diet (healthy), n=445	0	0	0	0	0	0	0	0	0	0	0	0	0	0	0	0	0	0	0	0	0	0	0
Maternal diseases (yes), n=801	0.29	0	0.06	0	0	0	0	0	0	0	0	0	0	0	0	0	0	0	0	0	0	0	0
Socio-economic disadvantage, n=705[2]	0	0	0	0	0	0	0	-0.08	0	0	0	0	0	0	0	0	0	0	0	0	0	0	0

Estimate				
	≤-1.0	-0.5	0.5	≥1.0

[1]Selected a priori to the HMO models

[2]750 m x 750 m grid size in the residential area, only included in additional analyses

[3]Reference group is partial breastfeeding

Supplementary table 2. Correlations between residential green environment variables (standardized, 750 m x 750 m grids).

N = 795	Pearson Correlation Coefficients		
	zNDVI	zVCDI	zNI
zNDVI	1	0.59***	0.80***
zVCDI0	0.59***	1	0.55***
zNI	0.80***	0.55***	1

*** p<0.001 ** p<0.01 *p<0.05

Supplementary table 3a. Normalized Difference Vegetation Index, NDVI, and interactions with secretor status and curvilinear associations (750 m x 750 m grids). Adjusted standardized estimates (95% confidence interval) in HMO diversity, individual HMO concentrations and HMO structural groups (nmol/mL) per one SD increase in greenness separately for non-secretors and secretors from the interaction and from the curvilinear association. Standardization means that both the NDVI and outcome have been standardized (log scale in all other outcome variables expect Diversity, which is in the original scale).

N=772	NDVI*Secretor-interaction			Curvilinear NDVI	
	Non-secretors	Secretors	P-value		P-value
	Est. (95 % CL)	Est. (95 % CL)		Est. (95 % CL)	
Diversity	0.035 (-0.144, 0.215)	0.049 (-0.028, 0.125)	0.89	0.029 (-0.029, 0.087)	0.33
Sum of HMOs	-0.005 (-0.068, 0.058)	0.000 (-0.029, 0.024)	0.89	-0.015 (-0.034, 0.010)	0.24
HMO-bound sialic acid	0.028 (-0.130, 0.183)	0.042 (-0.025, 0.106)	0.88	-0.004 (-0.053, 0.049)	0.95
HMO-bound fucose	-0.039 (-0.113, 0.034)	0.008 (-0.024, 0.039)	0.25	0.003 (-0.021, 0.026)	0.75
2'FL	-0.089 (-0.139, -0.039)	-0.010 (-0.030, 0.012)	**0.004**	-0.004 (-0.020, 0.012)	0.59
3FL	0.030 (-0.105, 0.166)	-0.008 (-0.065, 0.049)	0.61	0.026 (-0.018, 0.070)	0.24
LNnT	0.134 (-0.045, 0.315)	0.052 (-0.025, 0.129)	0.41	0.015 (-0.045, 0.072)	0.62
3'SL	0.059 (-0.118, 0.237)	0.008 (-0.068, 0.085)	0.60	0.006 (-0.051, 0.063)	0.82
DFLac	0.077 (0.004, 0.150)	0.013 (-0.019, 0.044)	0.11	0.011 (-0.012, 0.035)	0.35
6'SL	-0.078 (-0.244, 0.090)	0.019 (-0.052, 0.090)	0.29	0.017 (-0.038, 0.071)	0.55
LNT	0.039 (-0.138, 0.217)	0.012 (-0.063, 0.087)	0.79	-0.022 (-0.079, 0.036)	0.44
LNFP I	-0.061 (-0.162, 0.039)	0.010 (-0.034, 0.053)	0.20	-0.010 (-0.042, 0.023)	0.55
LNFP II	-0.066 (-0.226, 0.094)	0.062 (-0.006, 0.130)	0.14	0.021 (-0.030, 0.072)	0.40
LNFP III	0.041 (-0.125, 0.204)	-0.042 (-0.112, 0.028)	0.37	0.022 (-0.031, 0.075)	0.41
LSTb	0.006 (-0.166, 0.177)	0.067 (-0.006, 0.139)	0.52	-0.004 (-0.060, 0.052)	0.89
LSTc	0.078 (-0.097, 0.252)	0.002 (-0.072, 0.076)	0.44	0.009 (-0.048, 0.065)	0.74
DFLNT	-0.042 (-0.213, 0.130)	0.087 (0.014, 0.160)	0.17	0.023 (-0.032, 0.079)	0.41
LNH	0.011 (-0.172, 0.194)	-0.022 (-0.099, 0.057)	0.74	-0.003 (-0.061, 0.057)	0.93
DSLNT	0.086 (-0.094, 0.266)	0.057 (-0.020, 0.135)	0.78	-0.012 (-0.068, 0.047)	0.71
FLNH	-0.190 (-0.362, -0.019)	0.005 (-0.069, 0.078)	**0.038**	-0.022 (-0.078, 0.034)	0.43
DFLNH	-0.096 (-0.248, 0.057)	0.038 (-0.026, 0.103)	0.11	0.014 (-0.035, 0.063)	0.59
FDSLNH	0.038 (-0.124, 0.199)	0.011 (-0.056, 0.080)	0.77	-0.020 (-0.072, 0.031)	0.43
DSLNH	0.101 (-0.077, 0.276)	-0.043 (-0.118, 0.032)	0.14	-0.037 (-0.094, 0.020)	0.21

Small HMOs	-0.073 (-0.158, 0.012)	-0.020 (-0.056, 0.017)	0.25	-0.020 (-0.046, 0.007)	0.16
Type 1 HMOs	0.081 (-0.076, 0.238)	0.019 (-0.048, 0.088)	0.48	-0.024 (-0.076, 0.026)	0.34
Type 2 HMOs	0.156 (-0.026, 0.334)	0.046 (-0.032, 0.121)	0.27	0.020 (-0.037, 0.078)	0.50
α-1-2-fucosylated HMOs	-0.061 (-0.111, -0.011)	-0.011 (-0.032, 0.011)	0.063	-0.007 (-0.023, 0.009)	0.38
Terminal α-2-6-sialylated HMOs	-0.082 (-0.247, 0.086)	0.020 (-0.050, 0.092)	0.26	0.020 (-0.034, 0.074)	0.46
Internal α-2-6-sialylated HMOs	0.066 (-0.112, 0.245)	0.062 (-0.012, 0.137)	0.96	-0.012 (-0.071, 0.044)	0.66
Terminal α-2-3-sialylated HMOs	0.108 (-0.067, 0.286)	0.032 (-0.044, 0.108)	0.43	0.003 (-0.055, 0.058)	0.98

Supplementary table 3b. Vegetation Cover Diversity, VCDI, and interactions with secretor status and curvilinear associations (750 m x 750 m grids). Adjusted standardized estimates (95% confidence interval) in HMO diversity, individual HMO concentrations and HMO structural groups (nmol/mL) per one SD increase in vegetation cover diversity separately for non-secretors and secretors from the interaction and from the curvilinear association. Standardization means that both the VCDI and outcome have been standardized (log scale in all other outcome variables expect Diversity, which is in the original scale).

N=772	VCDI*Secretor-interaction			Curvilinear VCDI	
	Non-secretors	Secretors	P-value		P-value
	Est. (95 % CL)	Est. (95 % CL)		Est. (95 % CL)	
Diversity	0.044 (-0.141, 0.230)	0.092 (0.016, 0.167)	0.65	0.048 (-0.012, 0.107)	0.11
Sum of HMOs	-0.019 (-0.087, 0.044)	-0.019 (-0.044, 0.010)	0.97	-0.015 (-0.034, 0.010)	0.23
HMO-bound sialic acid	-0.056 (-0.218, 0.106)	0.025 (-0.039, 0.092)	0.35	0.007 (-0.046, 0.060)	0.78
HMO-bound fucose	0.016 (-0.060, 0.092)	-0.008 (-0.039, 0.024)	0.57	-0.003 (-0.029, 0.021)	0.78
2'FL	-0.083 (-0.134, -0.031)	-0.017 (-0.037, 0.004)	**0.020**	0.001 (-0.016, 0.017)	0.99
3FL	0.051 (-0.089, 0.190)	-0.029 (-0.086, 0.029)	0.30	0.013 (-0.032, 0.057)	0.57
LNnT	0.065 (-0.122, 0.258)	0.062 (-0.012, 0.139)	0.98	0.015 (-0.045, 0.075)	0.61
3'SL	0.046 (-0.135, 0.230)	-0.046 (-0.120, 0.027)	0.35	0.015 (-0.042, 0.074)	0.60
DFLac	0.176 (0.101, 0.250)	0.009 (-0.022, 0.039)	**<0.0001**	-0.001 (-0.024, 0.024)	0.99
6'SL	-0.206 (-0.377, -0.035)	0.024 (-0.045, 0.093)	**0.015**	0.010 (-0.045, 0.064)	0.74
LNT	0.148 (-0.034, 0.331)	0.051 (-0.022, 0.126)	0.34	0.016 (-0.043, 0.073)	0.61
LNFP I	-0.055 (-0.16, 0.050)	0.008 (-0.035, 0.050)	0.28	0.013 (-0.02, 0.046)	0.44
LNFP II	-0.068 (-0.232, 0.096)	0.055 (-0.013, 0.121)	0.18	0.011 (-0.043, 0.064)	0.69
LNFP III	-0.011 (-0.180, 0.160)	-0.006 (-0.074, 0.064)	0.95	0.007 (-0.048, 0.063)	0.79
LSTb	0.027 (-0.148, 0.204)	0.119 (0.048, 0.191)	0.35	0.012 (-0.044, 0.067)	0.68
LSTc	0.155 (-0.026, 0.335)	-0.041 (-0.113, 0.034)	**0.050**	0.055 (-0.004, 0.113)	0.063
DFLNT	0.010 (-0.167, 0.186)	0.066 (-0.006, 0.137)	0.57	0.011 (-0.045, 0.067)	0.71
LNH	0.090 (-0.099, 0.279)	0.011 (-0.066, 0.087)	0.45	0.017 (-0.043, 0.077)	0.58
DSLNT	0.068 (-0.117, 0.254)	0.082 (0.008, 0.158)	0.89	0.014 (-0.047, 0.072)	0.67
FLNH	-0.146 (-0.323, 0.031)	0.043 (-0.030, 0.114)	0.054	0.019 (-0.037, 0.076)	0.49
DFLNH	0.009 (-0.147, 0.167)	0.038 (-0.024, 0.103)	0.73	0.015 (-0.035, 0.066)	0.55
FDSLNH	0.053 (-0.113, 0.219)	0.036 (-0.031, 0.103)	0.86	-0.022 (-0.075, 0.031)	0.42
DSLNH	0.128 (-0.055, 0.308)	-0.083 (-0.158, -0.010)	**0.035**	-0.002 (-0.060, 0.056)	0.94

Small HMOs	-0.139 (-0.226, -0.053)	-0.039 (-0.075, -0.003)	**0.037**	-0.022 (-0.049, 0.007)	0.12
Type 1 HMOs	0.098 (-0.064, 0.262)	0.050 (-0.017, 0.114)	0.59	0.024 (-0.029, 0.076)	0.38
Type 2 HMOs	0.095 (-0.089, 0.282)	0.055 (-0.020, 0.130)	0.69	0.026 (-0.035, 0.084)	0.40
α-1-2-fucosylated HMOs	-0.040 (-0.091, 0.012)	-0.018 (-0.039, 0.003)	0.45	0.000 (-0.017, 0.016)	0.97
Terminal α-2-6-sialylated HMOs	-0.207 (-0.378, -0.038)	0.018 (-0.050, 0.088)	**0.016**	0.018 (-0.038, 0.072)	0.54
Internal α-2-6-sialylated HMOs	0.062 (-0.12, 0.245)	0.095 (0.021, 0.170)	0.74	0.015 (-0.044, 0.073)	0.64
Terminal α-2-3-sialylated HMOs	0.096 (-0.087, 0.28)	-0.006 (-0.079, 0.070)	0.32	0.020 (-0.038, 0.079)	0.49

Supplementary table 3c. Naturalness Index, NI, and interactions with secretor status and curvilinear associations (750 m x 750 m grids). Adjusted standardized estimates (95% confidence interval) in HMO diversity, individual HMO concentrations and HMO structural groups (nmol/mL) per one SD increase in naturalness separately for non-secretors and secretors from the interaction and from the curvilinear association. Standardization means that both the NI and outcome have been standardized (log scale in all other outcome variables expect Diversity, which is in the original scale).

N=772	NI*Secretor-interaction			Curvilinear NI	
	Non-secretors	Secretors	P-value		P-value
	Est. (95 % CL)	Est. (95 % CL)		Est. (95 % CL)	
Diversity	0.065 (-0.144, 0.261)	0.106 (0.031, 0.180)	0.66	0.018 (-0.030, 0.067)	0.46
Sum of HMOs	-0.019 (-0.092, 0.053)	-0.019 (-0.044, 0.010)	0.97	0.005 (-0.015, 0.019)	0.90
HMO-bound sialic acid	0.007 (-0.169, 0.183)	0.067 (0.004, 0.130)	0.54	0.021 (-0.021, 0.063)	0.30
HMO-bound fucose	-0.008 (-0.092, 0.073)	-0.005 (-0.034, 0.026)	0.92	0.005 (-0.016, 0.024)	0.64
2'FL	-0.054 (-0.110, 0.002)	-0.020 (-0.041, 0.001)	0.27	-0.002 (-0.015, 0.012)	0.83
3FL	0.026 (-0.128, 0.179)	-0.014 (-0.070, 0.041)	0.63	0.038 (0.002, 0.075)	**0.042**
LNnT	0.151 (-0.052, 0.353)	0.050 (-0.025, 0.124)	0.36	-0.030 (-0.079, 0.017)	0.22
3'SL	0.044 (-0.156, 0.243)	-0.006 (-0.080, 0.068)	0.65	0.053 (0.006, 0.101)	**0.029**
DFLac	0.058 (-0.024, 0.141)	0.019 (-0.011, 0.049)	0.38	0.013 (-0.007, 0.032)	0.21
6'SL	-0.090 (-0.277, 0.097)	0.052 (-0.016, 0.121)	0.16	0.021 (-0.024, 0.066)	0.36
LNT	0.022 (-0.178, 0.223)	0.039 (-0.034, 0.112)	0.87	-0.041 (-0.089, 0.008)	0.10
LNFP I	-0.153 (-0.266, 0.039)	0.002 (-0.04, 0.044)	**0.012**	-0.028 (-0.055, -0.001)	**0.044**
LNFP II	0.006 (-0.172, 0.185)	0.051 (-0.015, 0.115)	0.65	0.002 (-0.043, 0.043)	0.96
LNFP III	-0.002 (-0.186, 0.184)	-0.035 (-0.105, 0.033)	0.74	0.006 (-0.039, 0.052)	0.80
LSTb	-0.025 (-0.217, 0.167)	0.112 (0.042, 0.183)	0.19	-0.021 (-0.067, 0.025)	0.38
LSTc	0.206 (0.011, 0.404)	-0.044 (-0.116, 0.028)	**0.018**	-0.005 (-0.051, 0.042)	0.84
DFLNT	-0.012 (-0.204, 0.182)	0.099 (0.029, 0.171)	0.29	0.017 (-0.029, 0.063)	0.47
LNH	-0.022 (-0.229, 0.183)	-0.025 (-0.101, 0.050)	0.98	-0.006 (-0.057, 0.044)	0.79
DSLNT	-0.031 (-0.232, 0.172)	0.096 (0.021, 0.170)	0.25	-0.020 (-0.068, 0.029)	0.43
FLNH	-0.248 (-0.442, -0.056)	0.028 (-0.043, 0.100)	**0.008**	0.012 (-0.035, 0.058)	0.63
DFLNH	0.012 (-0.159, 0.184)	0.003 (-0.061, 0.066)	0.92	-0.054 (-0.095, -0.014)	**0.010**
FDSLNH	0.088 (-0.094, 0.269)	-0.009 (-0.075, 0.058)	0.32	-0.030 (-0.072, 0.016)	0.19
DSLNH	0.201 (0.002, 0.399)	-0.055 (-0.126, 0.019)	**0.018**	-0.009 (-0.056, 0.039)	0.71

Small HMOs	-0.087 (-0.180, 0.008)	-0.037 (-0.071, -0.002)	0.34	0.014 (-0.010, 0.036)	0.27
Type 1 HMOs	-0.007 (-0.186, 0.172)	0.038 (-0.029, 0.103)	0.65	-0.052 (-0.093, -0.010)	**0.018**
Type 2 HMOs	0.158 (-0.043, 0.360)	0.037 (-0.037, 0.112)	0.27	-0.029 (-0.078, 0.020)	0.23
α-1-2-fucosylated HMOs	-0.085 (-0.142, -0.029)	-0.023 (-0.043, -0.002)	**0.039**	-0.008 (-0.021, 0.006)	0.25
Terminal α-2-6-sialylated HMOs	-0.094 (-0.281, 0.092)	0.048 (-0.022, 0.116)	0.16	0.022 (-0.024, 0.066)	0.36
Internal α-2-6-sialylated HMOs	-0.031 (-0.230, 0.168)	0.106 (0.033, 0.180)	0.20	-0.019 (-0.066, 0.029)	0.46
Terminal α-2-3-sialylated HMOs	0.009 (-0.192, 0.207)	0.047 (-0.029, 0.120)	0.73	0.041 (-0.006, 0.090)	0.092

Supplementary table 4. Adjusted log-estimates (95% confidence interval), prior to outcome standardization, in HMO diversity, individual HMO concentrations and HMO structural groups (nmol/mL) per one SD increase in residential green environment (exposure) variables (750 m x 750 m grids). Log scale in all other outcome variables expect Diversity, which is in the original scale. Statistics are given for main effects. ***** indicates a significant interaction between the exposure variable and secretor status (p<0.05). $^{\mathbf{C}}$ indicates a significant curvilinear association with the exposure variable (p<0.05). Models were adjusted for secretor status, season, lactation time, lactation status, child sex, child birth weight, birth mode, duration of pregnancy, number of previous births, marital status, occupation, education, smoking, pre-pregnancy BMI, diseases.

N=772	NDVI, Normalized Difference Vegetation Index		VCDI, Vegetation Cover Diversity		NI, Naturalness Index	
	Est. (95 % CL)	p-value	Est. (95 % CL)	p-value	Est. (95 % CL)	p-value
Diversity	0.068 (-0.042, 0.18)	0.23	0.130 (0.023, 0.237)	**0.017**	0.154 (0.046, 0.262)	**0.005**
Sum of HMOs	-0.0004 (-0.006, 0.005)	0.87	-0.004 (-0.010, 0.013)	0.13	-0.004 (-0.009, 0.001)	0.14
HMO-bound sialic acid	0.011 (-0.007, 0.028)	0.23	0.004 (-0.013, 0.021)	0.66	0.017 (-0.001, 0.034)	0.053
HMO-bound fucose	0.0005 (-0.011, 0.012)	0.92	-0.002 (-0.013, 0.009)	0.75	-0.002 (-0.013, 0.009)	0.76
2'FL	-0.035 (-0.069, -0.002)	**0.036***	-0.043 (-0.076, -0.011)	**0.010***	-0.041 (-0.073, -0.008)	**0.015**
3FL	-0.0006 (-0.034, 0.033)	0.97	-0.011 (-0.044, 0.022)	0.51	-0.006 (-0.039, 0.027)	0.72^{C}
LNnT	0.026 (-0.003, 0.055)	0.079	0.026 (-0.003, 0.054)	0.077	0.025 (-0.004, 0.053)	0.087
3'SL	0.0084 (-0.025, 0.042)	0.62	-0.016 (-0.048, 0.017)	0.35	-0.001 (-0.033, 0.033)	0.99^{C}
DFLac	0.035 (-0.012, 0.082)	0.14	0.052 (0.006, 0.098)	**0.026***	0.039 (-0.008, 0.085)	0.10
6'SL	0.003 (-0.035, 0.041)	0.87	-0.005 (-0.042, 0.033)	0.81*	0.021 (-0.017, 0.059)	0.28
LNT	0.007 (-0.028, 0.043)	0.69	0.033 (-0.001, 0.068)	0.060	0.019 (-0.016, 0.054)	0.29
LNFP I	-0.005 (-0.053, 0.042)	0.82	-0.002 (-0.049, 0.045)	0.94	-0.019 (-0.066, 0.028)	0.43*^{C}
LNFP II	0.021 (-0.008, 0.051)	0.15	0.017 (-0.012, 0.046)	0.24	0.021 (-0.008, 0.050)	0.16
LNFP III	-0.013 (-0.048, 0.022)	0.48	-0.003 (-0.038, 0.031)	0.85	-0.017 (-0.052, 0.018)	0.35
LSTb	0.028 (-0.007, 0.063)	0.12	0.055 (0.021, 0.089)	**0.002**	0.050 (0.016, 0.085)	**0.005**
LSTc	0.009 (-0.031, 0.045)	0.70	-0.007 (-0.046, 0.031)	0.72*	-0.009 (-0.048, 0.030)	0.65*
DFLNT	0.059 (0.002, 0.12)	**0.042**	0.048 (-0.007, 0.104)	0.089	0.073 (0.017, 0.129)	**0.011**
LNH	-0.009 (-0.055, 0.037)	0.70	0.014 (-0.031, 0.059)	0.54	-0.016 (-0.061, 0.029)	0.49
DSLNT	0.029 (-0.008, 0.065)	0.12	0.041 (0.006, 0.077)	**0.024**	0.042 (0.006, 0.078)	**0.023**
FLNH	-0.019 (-0.072, 0.033)	0.47*	0.012 (-0.040, 0.064)	0.65	-0.003 (-0.055, 0.049)	0.92*
DFLNH	0.012 (-0.028, 0.051)	0.56	0.023 (-0.016, 0.061)	0.25	0.002 (-0.037, 0.041)	0.91^{C}
FDSLNH	0.011 (-0.030, 0.052)	0.59	0.025 (-0.015, 0.065)	0.22	0.002 (-0.039, 0.042)	0.94

DSLNH	-0.011 (-0.052, 0.031)	0.60	-0.032 (-0.072, 0.008)	0.12*	-0.014 (-0.055, 0.026)	0.49*
Small HMOs	-0.016 (-0.036, 0.003)	0.11	-0.031 (-0.050, -0.012)	**0.002***	-0.025 (-0.044, -0.006)	**0.012**
Type 1 HMOs	0.012 (-0.014, 0.038)	0.37	0.024 (-0.002, 0.049)	0.074	0.014 (-0.012, 0.040)	0.31[C]
Type 2 HMOs	0.021 (-0.004, 0.046)	0.093	0.021 (-0.003, 0.045)	0.089	0.018 (-0.006, 0.042)	0.15
α-1-2-fucosylated HMOs	-0.025 (-0.053, 0.003)	0.077	-0.030 (-0.057, -0.002)	**0.033**	-0.042 (-0.069, -0.014)	**0.003***
Terminal α-2-6-sialylated HMOs	0.003 (-0.030, 0.036)	0.86	-0.007 (-0.039, 0.025)	0.68*	0.016 (-0.017, 0.048)	0.35
Internal α-2-6-sialylated HMOs	0.030 (-0.004, 0.064)	0.079	0.044 (0.011, 0.077)	**0.010**	0.044 (0.010, 0.077)	**0.010**
Terminal α-2-3-sialylated HMOs	0.015 (-0.009, 0.039)	0.22	0.003 (-0.020, 0.027)	0.79	0.014 (-0.010, 0.038)	0.24

Supplementary table 5a. Normalized Difference Vegetation Index, NDVI, and interactions with secretor status and curvilinear associations (750 m x 750 m grids). Adjusted log-estimates (95% confidence interval), prior to outcome standardization, in HMO diversity, individual HMO concentrations and HMO structural groups (nmol/mL) per one SD increase in greenness variable separately for non-secretors and secretors from the interaction and from the curvilinear association. Log scale in all other outcome variables expect Diversity, which is in the original scale.

N=772	NDVI*Secretor-interaction			Curvilinear NDVI	
	Non-secretors	Secretors	P-value		P-value
	Est. (95 % CL)	Est. (95 % CL)		Est. (95 % CL)	
Diversity	0.054 (-0.221, 0.330)	0.075 (-0.043, 0.191)	0.89	0.044 (-0.045, 0.133)	0.33
Sum of HMOs	-0.001 (-0.014, 0.012)	-0.000 (-0.006, 0.005)	0.89	-0.003 (-0.007, 0.002)	0.24
HMO-bound sialic acid	0.008 (-0.037, 0.052)	0.012 (-0.007, 0.030)	0.88	-0.001 (-0.015, 0.014)	0.95
HMO-bound fucose	-0.015 (-0.043, 0.013)	0.003 (-0.009, 0.015)	0.25	0.001 (-0.008, 0.010)	0.75
2'FL	-0.149 (-0.232, -0.066)	-0.016 (-0.051, 0.020)	**0.004**	-0.007 (-0.034, 0.020)	0.59
3FL	0.019 (-0.066, 0.104)	-0.005 (-0.041, 0.031)	0.61	0.016 (-0.011, 0.044)	0.24
LNnT	0.054 (-0.018, 0.127)	0.021 (-0.010, 0.052)	0.41	0.006 (-0.018, 0.029)	0.62
3'SL	0.028 (-0.056, 0.112)	0.004 (-0.032, 0.040)	0.60	0.003 (-0.024, 0.030)	0.82
DFLac	0.124 (0.006, 0.243)	0.021 (-0.030, 0.071)	0.11	0.018 (-0.020, 0.057)	0.35
6'SL	-0.045 (-0.141, 0.052)	0.011 (-0.030, 0.052)	0.29	0.010 (-0.022, 0.041)	0.55
LNT	0.020 (-0.070, 0.110)	0.006 (-0.032, 0.044)	0.79	-0.011 (-0.040, 0.018)	0.44
LNFP I	-0.073 (-0.193, 0.046)	0.012 (-0.040, 0.063)	0.20	-0.012 (-0.050, 0.027)	0.55
LNFP II	-0.031 (-0.106, 0.044)	0.029 (-0.003, 0.061)	0.14	0.010 (-0.014, 0.034)	0.40
LNFP III	0.022 (-0.068, 0.111)	-0.023 (-0.061, 0.015)	0.37	0.012 (-0.017, 0.041)	0.41
LSTb	0.003 (-0.086, 0.092)	0.035 (-0.003, 0.072)	0.52	-0.002 (-0.031, 0.027)	0.89
LSTc	0.044 (-0.055, 0.143)	0.001 (-0.041, 0.043)	0.44	0.005 (-0.027, 0.037)	0.74
DFLNT	-0.035 (-0.178, 0.109)	0.073 (0.012, 0.134)	0.17	0.019 (-0.027, 0.066)	0.41
LNH	0.007 (-0.109, 0.123)	-0.014 (-0.063, 0.036)	0.74	-0.002 (-0.039, 0.036)	0.93
DSLNT	0.044 (-0.048, 0.136)	0.029 (-0.010, 0.069)	0.78	-0.006 (-0.035, 0.024)	0.71
FLNH	-0.147 (-0.280, -0.015)	0.004 (-0.053, 0.060)	**0.038**	-0.017 (-0.060, 0.026)	0.43
DFLNH	-0.063 (-0.162, 0.037)	0.025 (-0.017, 0.067)	0.11	0.009 (-0.023, 0.041)	0.59
FDSLNH	0.024 (-0.079, 0.127)	0.007 (-0.036, 0.051)	0.77	-0.013 (-0.046, 0.020)	0.43
DSLNH	0.059 (-0.045, 0.162)	-0.025 (-0.069, 0.019)	0.14	-0.022 (-0.055, 0.012)	0.21
Small HMOs	-0.043 (-0.093, 0.007)	-0.012 (-0.033, 0.010)	0.25	-0.012 (-0.027, 0.004)	0.16
Type 1 HMOs	0.034 (-0.032, 0.100)	0.008 (-0.020, 0.037)	0.48	-0.010 (-0.032, 0.011)	0.34

Type 2 HMOs	0.054 (-0.009, 0.116)	0.016 (-0.011, 0.042)	0.27	0.007 (-0.013, 0.027)	0.50
α-1-2-fucosylated HMOs	-0.086 (-0.156, -0.016)	-0.015 (-0.045, 0.015)	0.063	-0.010 (-0.033, 0.012)	0.38
Terminal α-2-6-sialylated HMOs	-0.041 (-0.124, 0.043)	0.010 (-0.025, 0.046)	0.26	0.010 (-0.017, 0.037)	0.46
Internal α-2-6-sialylated HMOs	0.032 (-0.054, 0.118)	0.030 (-0.006, 0.066)	0.96	-0.006 (-0.034, 0.021)	0.66
Terminal α-2-3-sialylated HMOs	0.037 (-0.023, 0.098)	0.011 (-0.015, 0.037)	0.43	0.001 (-0.019, 0.020)	0.98

Supplementary table 5b. Vegetation Cover Diversity, VCDI, and interactions with secretor status and curvilinear associations (750 m x 750 m grids). Adjusted log-estimates (95% confidence interval), prior to outcome standardization, in HMO diversity, individual HMO concentrations and HMO structural groups (nmol/mL) per one SD increase in vegetation cover diversity variables separately for non-secretors and secretors from the interaction and from the curvilinear association. Log scale in all other outcome variables expect Diversity, which is in the original scale.

N=772	VCDI*Secretor-interaction			Curvilinear VCDI	
	Non-secretors	Secretors	P-value		P-value
	Est. (95 % CL)	Est. (95 % CL)		Est. (95 % CL)	
Diversity	0.068 (-0.216, 0.353)	0.141 (0.025, 0.256)	0.65	0.073 (-0.018, 0.164)	0.11
Sum of HMOs	-0.004 (-0.018, 0.009)	-0.004 (-0.009, 0.002)	0.97	-0.003 (-0.007, 0.002)	0.23
HMO-bound sialic acid	-0.016 (-0.062, 0.030)	0.007 (-0.011, 0.026)	0.35	0.002 (-0.013, 0.017)	0.78
HMO-bound fucose	0.006 (-0.023, 0.035)	-0.003 (-0.015, 0.009)	0.57	-0.001 (-0.011, 0.008)	0.78
2'FL	-0.138 (-0.224, -0.052)	-0.028 (-0.062, 0.007)	**0.020**	0.001 (-0.027, 0.028)	0.99
3FL	0.032 (-0.056, 0.119)	-0.018 (-0.054, 0.018)	0.30	0.008 (-0.020, 0.036)	0.57
LNnT	0.026 (-0.049, 0.104)	0.025 (-0.005, 0.056)	0.98	0.006 (-0.018, 0.030)	0.61
3'SL	0.022 (-0.064, 0.109)	-0.022 (-0.057, 0.013)	0.35	0.007 (-0.020, 0.035)	0.60
DFLac	0.285 (0.164, 0.405)	0.014 (-0.036, 0.063)	**<0.0001**	-0.001 (-0.039, 0.039)	0.99
6'SL	-0.119 (-0.218, -0.020)	0.014 (-0.026, 0.054)	**0.015**	0.006 (-0.026, 0.037)	0.74
LNT	0.075 (-0.017, 0.168)	0.026 (-0.011, 0.064)	0.34	0.008 (-0.022, 0.037)	0.61
LNFP I	-0.066 (-0.190, 0.059)	0.009 (-0.042, 0.059)	0.28	0.016 (-0.024, 0.055)	0.44
LNFP II	-0.032 (-0.109, 0.045)	0.026 (-0.006, 0.057)	0.18	0.005 (-0.020, 0.030)	0.69
LNFP III	-0.006 (-0.098, 0.087)	-0.003 (-0.040, 0.035)	0.95	0.004 (-0.026, 0.034)	0.79
LSTb	0.014 (-0.077, 0.106)	0.062 (0.025, 0.099)	0.35	0.006 (-0.023, 0.035)	0.68
LSTc	0.088 (-0.015, 0.190)	-0.023 (-0.064, 0.019)	**0.050**	0.031 (-0.002, 0.064)	0.063
DFLNT	0.008 (-0.140, 0.156)	0.055 (-0.005, 0.115)	0.57	0.009 (-0.038, 0.056)	0.71
LNH	0.057 (-0.063, 0.177)	0.007 (-0.042, 0.055)	0.45	0.011 (-0.027, 0.049)	0.58
DSLNT	0.035 (-0.060, 0.130)	0.042 (0.004, 0.081)	0.89	0.007 (-0.024, 0.037)	0.67
FLNH	-0.113 (-0.250, 0.024)	0.033 (-0.023, 0.088)	0.054	0.015 (-0.029, 0.059)	0.49
DFLNH	0.006 (-0.096, 0.109)	0.025 (-0.016, 0.067)	0.73	0.010 (-0.023, 0.043)	0.55
FDSLNH	0.034 (-0.072, 0.140)	0.023 (-0.020, 0.066)	0.86	-0.014 (-0.048, 0.020)	0.42
DSLNH	0.075 (-0.032, 0.181)	-0.049 (-0.093, -0.006)	**0.035**	-0.001 (-0.035, 0.033)	0.94
Small HMOs	-0.082 (-0.133, -0.031)	-0.023 (-0.044, -0.002)	**0.037**	-0.013 (-0.029, 0.004)	0.12
Type 1 HMOs	0.041 (-0.027, 0.110)	0.021 (-0.007, 0.048)	0.59	0.010 (-0.012, 0.032)	0.38

Type 2 HMOs	0.033 (-0.031, 0.098)	0.019 (-0.007, 0.045)	0.69	0.009 (-0.012, 0.029)	0.40
α-1-2-fucosylated HMOs	-0.056 (-0.128, 0.017)	-0.025 (-0.055, 0.004)	0.45	-0.000 (-0.024, 0.023)	0.97
Terminal α-2-6-sialylated HMOs	-0.104 (-0.190, -0.019)	0.009 (-0.025, 0.044)	**0.016**	0.009 (-0.019, 0.036)	0.54
Internal α-2-6-sialylated HMOs	0.030 (-0.058, 0.118)	0.046 (0.010, 0.082)	0.74	0.007 (-0.021, 0.035)	0.64
Terminal α-2-3-sialylated HMOs	0.033 (-0.030, 0.096)	-0.002 (-0.027, 0.024)	0.32	0.007 (-0.013, 0.027)	0.49

Supplementary table 5c. Naturalness Index, NI, and interactions with secretor status and curvilinear associations (750 m x 750 m grids). Adjusted log-estimates (95% confidence interval), prior to outcome standardization, in HMO diversity, individual HMO concentrations and HMO structural groups (nmol/mL) per one SD increase in naturalness separately for non-secretors and secretors from the interaction and from the curvilinear association. Log scale in all other outcome variables expect Diversity, which is in the original scale.

N=772	NI*Secretor-interaction			Curvilinear NI	
	Non-secretors	Secretors	P-value		P-value
	Est. (95 % CL)	Est. (95 % CL)		Est. (95 % CL)	
Diversity	0.099 (-0.220, 0.400)	0.162 (0.048, 0.276)	0.66	0.028 (-0.046, 0.102)	0.46
Sum of HMOs	-0.004 (-0.019, 0.011)	-0.004 (-0.009, 0.002)	0.97	0.001 (-0.003, 0.004)	0.90
HMO-bound sialic acid	0.002 (-0.048, 0.052)	0.019 (0.001, 0.037)	0.54	0.006 (-0.006, 0.018)	0.30
HMO-bound fucose	-0.003 (-0.035, 0.028)	-0.002 (-0.013, 0.010)	0.92	0.002 (-0.006, 0.009)	0.64
2'FL	-0.090 (-0.184, 0.004)	-0.034 (-0.069, 0.001)	0.27	-0.003 (-0.025, 0.020)	0.83
3FL	0.016 (-0.080, 0.112)	-0.009 (-0.044, 0.026)	0.63	0.024 (0.001, 0.047)	**0.042**
LNnT	0.061 (-0.021, 0.142)	0.020 (-0.010, 0.050)	0.36	-0.012 (-0.032, 0.007)	0.22
3'SL	0.021 (-0.074, 0.115)	-0.003 (-0.038, 0.032)	0.65	0.025 (0.003, 0.048)	**0.029**
DFLac	0.094 (-0.039, 0.228)	0.031 (-0.018, 0.080)	0.38	0.021 (-0.012, 0.052)	0.21
6'SL	-0.052 (-0.160, 0.056)	0.030 (-0.009, 0.070)	0.16	0.012 (-0.014, 0.038)	0.36
LNT	0.011 (-0.090, 0.113)	0.020 (-0.017, 0.057)	0.87	-0.021 (-0.045, 0.004)	0.10
LNFP I	-0.182 (-0.317, -0.047)	0.002 (-0.048, 0.052)	**0.012**	-0.033 (-0.066, -0.001)	**0.044**
LNFP II	0.003 (-0.081, 0.087)	0.024 (-0.007, 0.054)	0.65	0.001 (-0.020, 0.021)	0.96
LNFP III	-0.001 (-0.101, 0.100)	-0.019 (-0.056, 0.018)	0.74	0.003 (-0.021, 0.027)	0.80
LSTb	-0.013 (-0.113, 0.087)	0.058 (0.022, 0.095)	0.19	-0.011 (-0.035, 0.013)	0.38
LSTc	0.117 (0.006, 0.229)	-0.025 (-0.066, 0.016)	**0.018**	-0.003 (-0.029, 0.024)	0.84
DFLNT	-0.010 (-0.171, 0.152)	0.083 (0.024, 0.143)	0.29	0.014 (-0.024, 0.053)	0.47
LNH	-0.014 (-0.145, 0.116)	-0.016 (-0.064, 0.032)	0.98	-0.004 (-0.036, 0.027)	0.79
DSLNT	-0.016 (-0.119, 0.088)	0.049 (0.011, 0.087)	0.25	-0.010 (-0.035, 0.015)	0.43
FLNH	-0.192 (-0.342, -0.043)	0.022 (-0.033, 0.077)	**0.008**	0.009 (-0.027, 0.045)	0.63
DFLNH	0.008 (-0.104, 0.120)	0.002 (-0.040, 0.043)	0.92	-0.035 (-0.062, -0.009)	**0.010**
FDSLNH	0.056 (-0.060, 0.172)	-0.006 (-0.048, 0.037)	0.32	-0.019 (-0.046, 0.010)	0.19
DSLNH	0.118 (0.001, 0.234)	-0.032 (-0.074, 0.011)	**0.018**	-0.005 (-0.033, 0.023)	0.71
Small HMOs	-0.051 (-0.106, 0.005)	-0.022 (-0.042, -0.001)	0.34	0.008 (-0.006, 0.021)	0.27
Type 1 HMOs	-0.003 (-0.078, 0.072)	0.016 (-0.012, 0.043)	0.65	-0.022 (-0.039, -0.004)	**0.018**

Type 2 HMOs	0.055 (-0.016, 0.125)	0.013 (-0.013, 0.039)	0.27	-0.010 (-0.027, 0.007)	0.23
α-1-2-fucosylated HMOs	-0.120 (-0.20, -0.041)	-0.032 (-0.061, -0.003)	**0.039**	-0.011 (-0.030, 0.008)	0.25
Terminal α-2-6-sialylated HMOs	-0.047 (-0.141, 0.046)	0.024 (-0.011, 0.058)	0.16	0.011 (-0.012, 0.033)	0.36
Internal α-2-6-sialylated HMOs	-0.015 (-0.111, 0.081)	0.051 (0.016, 0.087)	0.20	-0.009 (-0.032, 0.014)	0.46
Terminal α-2-3-sialylated HMOs	0.003 (-0.066, 0.071)	0.016 (-0.010, 0.041)	0.73	0.014 (-0.002, 0.031)	0.092

Supplementary table 6a. Secretor status adjusted standardized estimates (95% confidence interval) in HMO diversity, individual HMO concentrations and HMO structural groups (nmol/mL) per one SD increase in residential green environment (exposure) variables. Standardization means that both the exposure and outcome have been standardized (log scale in all other outcome variables expect Diversity, which is in the original scale). The exposure variables were measured at the time of child was born (2008-2010) with 750 m x 750 m grid size around the homes of the families. Statistics are given for main effects. * indicates a significant interaction between the exposure variable and secretor status (p<0.05). C indicates a significant curvilinear association with the exposure variable (p<0.05). Models were adjusted only for secretor status.

N=772	NDVI, Normalized Difference Vegetation Index		VCDI, Vegetation Cover Diversity		NI, Naturalness Index	
	Est. (95 % CL)	p-value	Est. (95 % CL)	p-value	Est. (95 % CL)	p-value
Diversity	0.047 (-0.022, 0.116)	0.19	0.089 (0.020, 0.157)	**0.012**	0.091 (0.023, 0.163)	**0.009**
Sum of HMOs	-0.004 (-0.029, 0.019)	0.73	-0.024 (-0.049, 0.005)	0.064	-0.019 (-0.044, 0.005)	0.16
HMO-bound sialic acid	0.025 (-0.039, 0.085)	0.46	0.011 (-0.049, 0.070)	0.73	0.053 (-0.011, 0.113)	0.094
HMO-bound fucose	-0.001 (-0.029, 0.029)	0.95	-0.005 (-0.034, 0.021)	0.66	-0.005 (-0.034, 0.024)	0.70
2'FL	-0.020 (-0.039, -0.001)	**0.036***	-0.026 (-0.045, -0.007)	**0.007***	-0.024 (-0.043, -0.005)	**0.014**
3FL	-0.016 (-0.069, 0.038)	0.56	-0.030 (-0.083, 0.024)	0.27	-0.018 (-0.072, 0.035)	0.50^{C}
LNnT	0.070 (-0.002, 0.139)	0.054	0.060 (-0.010, 0.129)	0.092	0.062 (-0.010, 0.132)	0.086
3'SL	0.006 (-0.061, 0.076)	0.84	-0.030 (-0.097, 0.040)	0.40	0.002 (-0.068, 0.070)	0.95^{C}
DFLac	0.015 (-0.012, 0.043)	0.28	0.030 (0.002, 0.057)	**0.034***	0.022 (-0.006, 0.050)	0.12
6'SL	-0.001 (-0.066, 0.062)	0.97	-0.017 (-0.081, 0.045)	0.58*	0.028 (-0.036, 0.092)	0.39
LNT	0.018 (-0.051, 0.087)	0.61	0.069 (0.002, 0.138)	**0.049**	0.036 (-0.034, 0.103)	0.32
LNFP I	-0.003 (-0.041, 0.036)	0.89	-0.004 (-0.043, 0.034)	0.82	-0.020 (-0.059, 0.018)	0.31^{*C}
LNFP II	0.047 (-0.015, 0.106)	0.14	0.036 (-0.026, 0.096)	0.25	0.043 (-0.019, 0.104)	0.17
LNFP III	-0.037 (-0.099, 0.026)	0.25	-0.015 (-0.077, 0.048)	0.65	-0.037 (-0.099, 0.026)	0.25
LSTb	0.056 (-0.010, 0.123)	0.095	0.102 (0.037, 0.167)	**0.002**	0.083 (0.017, 0.148)	**0.014**
LSTc	0.001 (-0.067, 0.071)	0.97	-0.021 (-0.090, 0.048)	0.56*	-0.014 (-0.083, 0.055)	0.70*
DFLNT	0.074 (0.008, 0.140)	**0.027**	0.066 (-0.001, 0.131)	0.051	0.088 (0.023, 0.154)	**0.009**
LNH	-0.008 (-0.077, 0.063)	0.84	0.022 (-0.047, 0.093)	0.52	-0.016 (-0.085, 0.054)	0.66
DSLNT	0.047 (-0.023, 0.115)	0.19	0.082 (0.014, 0.150)	**0.020**	0.066 (-0.004, 0.135)	0.063
FLNH	-0.019 (-0.087, 0.047)	0.57*	0.014 (-0.052, 0.081)	0.67	-0.003 (-0.069, 0.065)	0.95*
DFLNH	0.011 (-0.047, 0.069)	0.73	0.024 (-0.034, 0.083)	0.42	-0.002 (-0.060, 0.057)	0.95^{C}
FDSLNH	0.030 (-0.033, 0.091)	0.35	0.042 (-0.019, 0.103)	0.18	0.016 (-0.047, 0.077)	0.63

DSLNH	-0.043 (-0.112, 0.026)	0.23	-0.055 (-0.123, 0.014)	0.12*	-0.027 (-0.097, 0.041)	0.43*
Small HMOs	-0.025 (-0.058, 0.007)	0.12	-0.054 (-0.087, -0.022)	**0.001***	-0.036 (-0.070, -0.003)	**0.029**
Type 1 HMOs	0.019 (-0.041, 0.081)	0.53	0.052 (-0.010, 0.112)	0.094	0.019 (-0.041, 0.081)	0.53[C]
Type 2 HMOs	0.063 (-0.006, 0.133)	0.072	0.058 (-0.012, 0.127)	0.11	0.052 (-0.017, 0.121)	0.14
α-1-2-fucosylated HMOs	-0.018 (-0.037, 0.001)	0.067	-0.022 (-0.041, -0.004)	**0.021**	-0.029 (-0.048, -0.010)	**0.003***
Terminal α-2-6-sialylated HMOs	-0.002 (-0.066, 0.062)	0.97	-0.024 (-0.088, 0.040)	0.45*	0.026 (-0.038, 0.090)	0.44
Internal α-2-6-sialylated HMOs	0.052 (-0.017, 0.120)	0.14	0.091 (0.023, 0.160)	**0.009**	0.075 (0.006, 0.143)	**0.032**
Terminal α-2-3-sialylated HMOs	0.026 (-0.044, 0.096)	0.46	0.015 (-0.055, 0.085)	0.69	0.035 (-0.035, 0.102)	0.33[C]

2'-fucosyllactose (2'FL), 3-fucosyllactose (3FL), lacto-N-neotetraose (LNnT), 3'-sialyllactose (3'SL), difucosyllactose (DFlac), 6'-sialyllactose (6'SL), lacto-N-tetraose (LNT), lacto-Nfucopentaose (LNFP) I, LNFP II, LNFP III, sialyl-LNT (LST) b, LSTc, difucosyllacto-LNT (DFLNT), lacto-N-hexaose (LNH), disialyllacto-N-tetraose (DSLNT), fucosyllacto-Nhexaose (FLNH), difucosyllacto-N-hexaose (DFLNH), fucodisialyllacto-lacto-N-hexaose (FDSLNH) and disialyllacto-N-hexaose (DSLNH). HMO groups: small HMOs (2'FL, 3FL, 3'SL, 6'SL, and DFLac), type 1 HMOs (LNT, LNFP I, LNFP II, LSTb, DSLNT), type 2 HMOs (LNnT, LNFP III, LSTc), α-1-2-fucosylated HMOs (2'FL, LNFP I), terminal α-2-6-sialylated HMOs (6'SL, LSTc), internal α-2-6-sialylated HMOs (DSLNT, LSTb), terminal α-2-3-sialylated HMOs (3'SL, DSLNT)

Supplementary table 6b. Secretor status adjusted log-estimates (95% confidence interval), prior to outcome standardization, in HMO diversity, individual HMO concentrations and HMO structural groups (nmol/mL) per one SD increase in residential green environment (exposure) variables (750 m x 750 m grids). Log scale in all other outcome variables expect Diversity, which is in the original scale. Statistics are given for main effects. * indicates a significant interaction between the exposure variable and secretor status (p<0.05). ^{C} indicates a significant curvilinear association with the exposure variable (p<0.05). Models were adjusted only for secretor status.

N=795	NDVI, Normalized Difference Vegetation Index		VCDI, Vegetation Cover Diversity		NI, Naturalness Index	
	Est. (95 % CL)	p-value	Est. (95 % CL)	p-value	Est. (95 % CL)	p-value
Diversity	0.072 (-0.034, 0.178)	0.19	0.136 (0.030, 0.24)	**0.012**	0.14 (0.036, 0.25)	**0.009**
Sum of HMOs	-0.0009 (-0.006, 0.004)	0.73	-0.005 (-0.010, 0.001)	0.064	-0.004 (-0.009, 0.001)	0.16
HMO-bound sialic acid	0.007 (-0.011, 0.024)	0.46	0.003 (-0.014, 0.020)	0.73	0.015 (-0.003, 0.032)	0.094
HMO-bound fucose	-0.0003 (-0.011, 0.011)	0.95	-0.002 (-0.013, 0.008)	0.66	-0.002 (-0.013, 0.009)	0.70
2'FL	-0.034 (-0.066, -0.002)	**0.036***	-0.044 (-0.075, -0.012)	**0.007***	-0.040 (-0.072, -0.008)	**0.014**
3FL	-0.0099 (-0.043, 0.024)	0.56	-0.019 (-0.052, 0.015)	0.27	-0.011 (-0.045, 0.022)	0.50^{C}
LNnT	0.028 (-0.001, 0.056)	0.054	0.024 (-0.004, 0.052)	0.092	0.025 (-0.004, 0.053)	0.086
3'SL	0.003 (-0.029, 0.036)	0.84	-0.014 (-0.046, 0.019)	0.40	0.0009 (-0.032, 0.033)	0.95^{C}
DFLac	0.025 (-0.020, 0.070)	0.28	0.048 (0.004, 0.093)	**0.034***	0.036 (-0.009, 0.081)	0.12
6'SL	-0.0006 (-0.038, 0.036)	0.97	-0.010 (-0.047, 0.026)	0.58*	0.016 (-0.021, 0.053)	0.39
LNT	0.009 (-0.026, 0.044)	0.61	0.035 (0.001, 0.070)	**0.049**	0.018 (-0.017, 0.052)	0.32
LNFP I	-0.003 (-0.049, 0.043)	0.89	-0.005 (-0.051, 0.040)	0.82	-0.024 (-0.070, 0.022)	0.31*^{C}
LNFP II	0.022 (-0.007, 0.050)	0.14	0.017 (-0.012, 0.045)	0.25	0.020 (-0.009, 0.049)	0.17
LNFP III	-0.020 (-0.054, 0.014)	0.25	-0.008 (-0.042, 0.026)	0.65	-0.020 (-0.054, 0.014)	0.25
LSTb	0.029 (-0.005, 0.064)	0.095	0.053 (0.019, 0.087)	**0.002**	0.043 (0.009, 0.077)	**0.014**
LSTc	0.0008 (-0.038, 0.040)	0.97	-0.012 (-0.051, 0.027)	0.56*	-0.008 (-0.047, 0.031)	0.70*
DFLNT	0.062 (0.007, 0.117)	**0.027**	0.055 (-0.001, 0.11)	0.051	0.074 (0.019, 0.129)	**0.009**
LNH	-0.005 (-0.049, 0.040)	0.84	0.014 (-0.030, 0.059)	0.52	-0.010 (-0.054, 0.034)	0.66
DSLNT	0.024 (-0.012, 0.059)	0.19	0.042 (0.007, 0.077)	**0.020**	0.034 (-0.002, 0.069)	0.063
FLNH	-0.015 (-0.067, 0.036)	0.57*	0.011 (-0.040, 0.063)	0.67	-0.002 (-0.053, 0.050)	0.95*
DFLNH	0.007 (-0.031, 0.045)	0.73	0.016 (-0.022, 0.054)	0.42	-0.001 (-0.039, 0.037)	0.95^{C}
FDSLNH	0.019 (-0.021, 0.058)	0.35	0.027 (-0.012, 0.066)	0.18	0.010 (-0.030, 0.049)	0.63
DSLNH	-0.025 (-0.066, 0.015)	0.23	-0.032 (-0.072, 0.008)	0.12*	-0.016 (-0.057, 0.024)	0.43*
Small HMOs	-0.015 (-0.034, 0.004)	0.12	-0.032 (-0.051, -0.013)	**0.001***	-0.021 (-0.041, -0.002)	**0.029**

Type 1 HMOs	0.008 (-0.017, 0.034)	0.53	0.022 (-0.004, 0.047)	0.094	0.008 (-0.017, 0.034)	0.53[C]
Type 2 HMOs	0.022 (-0.002, 0.046)	0.072	0.020 (-0.004, 0.044)	0.11	0.018 (-0.006, 0.042)	0.14
α-1-2-fucosylated HMOs	-0.025 (-0.052, 0.002)	0.067	-0.031 (-0.058, -0.005)	**0.021**	-0.041 (-0.067, -0.014)	**0.003***
Terminal α-2-6-sialylated HMOs	-0.001 (-0.033, 0.031)	0.97	-0.012 (-0.044, 0.020)	0.45*	0.013 (-0.019, 0.045)	0.44
Internal α-2-6-sialylated HMOs	0.025 (-0.008, 0.058)	0.14	0.044 (0.011, 0.077)	**0.009**	0.036 (0.003, 0.069)	**0.032**
Terminal α-2-3-sialylated HMOs	0.009 (-0.015, 0.033)	0.46	0.005 (-0.019, 0.029)	0.69	0.012 (-0.012, 0.035)	0.33[C]

Supplementary table 7a. Adjusted standardized estimates (95% confidence interval) in HMO diversity, individual HMO concentrations and HMO structural groups (nmol/mL) per one SD increase in residential green environment (exposure) variables. Standardization means that both the exposure and outcome have been standardized (log scale in all other outcome variables expect Diversity, which is in the original scale). **The exposure variables were measured at the time the child was born (2008-2010) with 250 m x 250 m grid size around the homes of the families.** Statistics are given for main effects. * indicates a significant interaction between the exposure variable and secretor status (p<0.05). [C] indicates a significant curvilinear association with the exposure variable (p<0.05). Models were adjusted for secretor status, season, lactation time, lactation status, child sex, child birth weight, birth mode, duration of pregnancy, number of previous births, marital status, occupation, education, smoking, pre-pregnancy BMI, diseases.

	NDVI, Normalized Difference Vegetation Index, n=772		VCDI, Vegetation Cover Diversity, n=760		NI, Naturalness Index, n=772	
	Est. (95 % CL)	p-value	Est. (95 % CL)	p-value	Est. (95 % CL)	p-value
Diversity	0.041 (-0.030, 0.112)	0.26	0.047 (-0.023, 0.117)	0.19	0.074 (0.005, 0.145)	**0.036**
Sum of HMOs	-0.010 (-0.034, 0.019)	0.55	-0.010 (-0.034, 0.015)	0.42	-0.015 (-0.039, 0.010)	0.18
HMO-bound sialic acid	0.042 (-0.021, 0.102)	0.18	0.007 (-0.053, 0.007)	0.79	0.046 (-0.014, 0.106)	0.13
HMO-bound fucose	0.010 (-0.018, 0.039)	0.43[C]	0.008 (-0.021, 0.037)	0.56	0.005 (-0.024, 0.034)	0.70
2'FL	-0.020 (-0.039, 0.001)	0.053*	-0.020 (-0.040, -0.001)	**0.042*[C]**	-0.012 (-0.032, -0.007)	0.23
3FL	0.019 (-0.033, 0.073)	0.47	0.011 (-0.041, 0.062)	0.69	0.016 (-0.037, 0.069)	0.54
LNnT	0.015 (-0.057, 0.084)	0.69	0.015 (-0.055, 0.084)	0.68	0.025 (-0.045, 0.097)	0.47
3'SL	0.051 (-0.019, 0.120)	0.15	-0.004 (-0.074, 0.063)	0.89	0.027 (-0.040, 0.097)	0.43[C]
DFLac	0.022 (-0.006, 0.051)	0.13	0.036 (0.008, 0.064)	**0.012***	0.013 (-0.015, 0.041)	0.37
6'SL	-0.012 (-0.078, 0.054)	0.72	-0.033 (-0.097, 0.033)	0.33	0.007 (-0.059, 0.071)	0.85
LNT	-0.006 (-0.075, 0.063)	0.87	0.032 (-0.036, 0.101)	0.36	0.010 (-0.059, 0.079)	0.79
LNFP I	-0.018 (-0.057, 0.023)	0.39	-0.015 (-0.054, 0.024)	0.45	-0.020 (-0.060, 0.018)	0.31*
LNFP II	0.047 (-0.015, 0.109)	0.14	0.034 (-0.028, 0.096)	0.28	0.040 (-0.021, 0.102)	0.21
LNFP III	-0.007 (-0.072, 0.057)	0.83	-0.004 (-0.068, 0.059)	0.89	-0.024 (-0.087, 0.041)	0.47
LSTb	0.050 (-0.017, 0.115)	0.15	0.085 (0.017, 0.150)	**0.014**	0.089 (0.021, 0.154)	**0.010**
LSTc	0.002 (-0.067, 0.071)	0.98	0.030 (-0.039, 0.097)	0.40	-0.046 (-0.113, 0.023)	0.19*
DFLNT	0.070 (0.002, 0.137)	**0.042**	0.033 (-0.033, 0.102)	0.32	0.062 (-0.004, 0.129)	0.065
LNH	-0.009 (-0.080, 0.063)	0.80	0.002 (-0.071, 0.073)	0.98	-0.035 (-0.106, 0.036)	0.34
DSLNT	0.041 (-0.029, 0.111)	0.25	0.055 (-0.016, 0.125)	0.12[C]	0.057 (-0.012, 0.127)	0.11
FLNH	-0.014 (-0.081, 0.053)	0.68	-0.005 (-0.072, 0.061)	0.87	-0.025 (-0.092, 0.041)	0.46

DFLNH	0.017 (-0.043, 0.077)	0.59	0.009 (-0.049, 0.067)	0.75	-0.032 (-0.077, 0.028)	0.30
FDSLNH	0.025 (-0.039, 0.088)	0.45	0.028 (-0.034, 0.089)	0.39	0.019 (-0.044, 0.081)	0.56
DSLNH	-0.022 (-0.090, 0.048)	0.55	-0.017 (-0.085, 0.051)	0.63*	-0.051 (-0.119, 0.017)	0.15*
Small HMOs	-0.025 (-0.058, 0.008)	0.14	-0.032 (-0.065, 0.002)	0.056*	-0.029 (-0.061, 0.005)	0.094*
Type 1 HMOs	0.002 (-0.064, 0.060)	0.96	0.024 (-0.036, 0.086)	0.43	0.010 (-0.052, 0.072)	0.76
Type 2 HMOs	0.014 (-0.058, 0.084)	0.71	0.014 (-0.055, 0.084)	0.67	0.009 (-0.060, 0.078)	0.80
α-1-2-fucosylated HMOs	-0.020 (-0.039, 0.001)	0.051	-0.019 (-0.039, 0.001)	0.052*	-0.021 (-0.041, -0.002)	**0.031***
Terminal α-2-6-sialylated HMOs	-0.010 (-0.076, 0.054)	0.76	-0.034 (-0.098, 0.032)	0.31	-0.004 (-0.068, 0.060)	0.91
Internal α-2-6-sialylated HMOs	0.046 (-0.025, 0.116)	0.20	0.066 (-0.002, 0.135)	0.061[C]	0.068 (-0.002, 0.137)	0.052
Terminal α-2-3-sialylated HMOs	0.064 (-0.006, 0.134)	0.071	0.017 (-0.052, 0.087)	0.63	0.05 (-0.020, 0.117)	0.17[C]

Supplementary table 7b. Adjusted log-estimates (95% confidence interval), prior to outcome standardization, in HMO diversity, individual HMO concentrations and HMO structural groups (nmol/mL) per one SD increase in residential greenness environment (exposure) variables. Log scale in all other outcome variables expect Diversity, which is in the original scale. **The exposure variables were measured at the time of child was born (2008-2010) with 250 m x 250 m grid size around the homes of the families.** Statistics are given for main effects. * indicates a significant interaction between the exposure variable and secretor status (p<0.05). ^{C} indicates a significant curvilinear association with the exposure variable (p<0.05). Models were adjusted for secretor status, season, lactation time, lactation status, child sex, child birth weight, birth mode, duration of pregnancy, number of previous births, marital status, occupation, education, smoking, pre-pregnancy BMI, diseases.

	NDVI, Normalized Difference Vegetation Index, n=772		VCDI, Vegetation Cover Diversity, n=760		NI, Naturalness Index, n=772	
	Est. (95 % CL)	p-value	Est. (95 % CL)	p-value	Est. (95 % CL)	p-value
Diversity	0.063 (-0.046, 0.171)	0.26	0.072 (-0.035, 0.180)	0.19	0.114 (0.008, 0.222)	**0.036**
Sum of HMOs	-0.002 (-0.007, 0.004)	0.55	-0.002 (-0.007, 0.003)	0.42	-0.003 (-0.008, 0.002)	0.18
HMO-bound sialic acid	0.012 (-0.006, 0.029)	0.18	0.002 (-0.015, 0.020)	0.79	0.013 (-0.004, 0.030)	0.13
HMO-bound fucose	0.004 (-0.007, 0.015)	0.43^{C}	0.003 (-0.008, 0.014)	0.56	0.002 (-0.009, 0.013)	0.70
2'FL	-0.033 (-0.066, 0.001)	0.053*	-0.034 (-0.067, -0.001)	**0.042***^{C}	-0.020 (-0.053, -0.012)	0.23
3FL	0.012 (-0.021, 0.046)	0.47	0.007 (-0.026, 0.039)	0.69	0.010 (-0.023, 0.043)	0.54
LNnT	0.006 (-0.023, 0.034)	0.69	0.006 (-0.022, 0.034)	0.68	0.010 (-0.018, 0.039)	0.47
3'SL	0.024 (-0.009, 0.057)	0.15	-0.002 (-0.035, 0.030)	0.89	0.013 (-0.019, 0.046)	0.43^{C}
DFLac	0.036 (-0.010, 0.083)	0.13	0.058 (0.013, 0.103)	**0.012***	0.021 (-0.025, 0.067)	0.37
6'SL	-0.007 (-0.045, 0.031)	0.72	-0.019 (-0.056, 0.019)	0.33	0.004 (-0.034, 0.041)	0.85
LNT	-0.003 (-0.038, 0.032)	0.87	0.016 (-0.018, 0.051)	0.36	0.005 (-0.030, 0.040)	0.79
LNFP I	-0.021 (-0.068, 0.027)	0.39	-0.018 (-0.064, 0.029)	0.45	-0.024 (-0.071, 0.022)	0.31*
LNFP II	0.022 (-0.007, 0.051)	0.14	0.016 (-0.013, 0.045)	0.28	0.019 (-0.010, 0.048)	0.21
LNFP III	-0.004 (-0.039, 0.031)	0.83	-0.002 (-0.037, 0.032)	0.89	-0.013 (-0.047, 0.022)	0.47
LSTb	0.026 (-0.009, 0.060)	0.15	0.044 (0.009, 0.078)	**0.014**	0.046 (0.011, 0.080)	**0.010**
LSTc	0.001 (-0.038, 0.040)	0.98	0.017 (-0.022, 0.055)	0.40	-0.026 (-0.064, 0.013)	0.19*
DFLNT	0.059 (0.002, 0.115)	**0.042**	0.028 (-0.028, 0.085)	0.32	0.052 (-0.003, 0.108)	0.065
LNH	-0.006 (-0.051, 0.040)	0.80	0.001 (-0.045, 0.046)	0.98	-0.022 (-0.067, 0.023)	0.34
DSLNT	0.021 (-0.015, 0.057)	0.25	0.028 (-0.008, 0.064)	0.12^{C}	0.029 (-0.006, 0.065)	0.11
FLNH	-0.011 (-0.063, 0.041)	0.68	-0.004 (-0.056, 0.047)	0.87	-0.019 (-0.071, 0.032)	0.46
DFLNH	0.011 (-0.028, 0.050)	0.59	0.006 (-0.032, 0.044)	0.75	-0.021 (-0.059, 0.018)	0.30
FDSLNH	0.016 (-0.025, 0.056)	0.45	0.018 (-0.022, 0.057)	0.39	0.012 (-0.028, 0.052)	0.56

DSLNH	-0.013 (-0.053, 0.028)	0.55	-0.010 (-0.050, 0.030)	0.63*	-0.030 (-0.070, 0.010)	0.15*
Small HMOs	-0.015 (-0.034, 0.005)	0.14	-0.019 (-0.038, 0.001)	0.056*	-0.017 (-0.036, 0.003)	0.094*
Type 1 HMOs	0.001 (-0.027, 0.025)	0.96	0.010 (-0.015, 0.036)	0.43	0.004 (-0.022, 0.030)	0.76
Type 2 HMOs	0.005 (-0.020, 0.029)	0.71	0.005 (-0.019, 0.029)	0.67	0.003 (-0.021, 0.027)	0.80
α-1-2-fucosylated HMOs	-0.028 (-0.055, 0.001)	0.051	-0.027 (-0.055, 0.001)	0.052*	-0.030 (-0.057, -0.003)	**0.031***
Terminal α-2-6-sialylated HMOs	-0.005 (-0.038, 0.027)	0.76	-0.017 (-0.049, 0.016)	0.31	-0.002 (-0.034, 0.030)	0.91
Internal α-2-6-sialylated HMOs	0.022 (-0.012, 0.056)	0.20	0.032 (-0.001, 0.065)	0.061[C]	0.033 (-0.001, 0.066)	0.052
Terminal α-2-3-sialylated HMOs	0.022 (-0.002, 0.046)	0.071	0.006 (-0.018, 0.030)	0.63	0.017 (-0.007, 0.040)	0.17[C]

Supplementary table 8a. Normalized Difference Vegetation Index, NDVI and interactions with secretor status and curvilinear associations (**250 m x 250 m grids**). Adjusted log-estimates (95% confidence interval) in HMO diversity, individual HMO concentrations and HMO structural groups (nmol/mL) per one SD increase in greenness separately for non-secretors and secretors from the interaction and from the curvilinear association. Log scale in all other outcome variables expect Diversity, which is in the original scale.

N=772	NDVI*Secretor-interaction			Curvilinear NDVI	
	Non-secretors	Secretors	P-value		P-value
	Est. (95 % CL)	Est. (95 % CL)		Est. (95 % CL)	
Diversity	0.061 (-0.214, 0.336)	0.064 (-0.053, 0.181)	0.99	0.024 (-0.064, 0.111)	0.60
Sum of HMOs	-0.004 (-0.017, 0.009)	-0.001 (-0.007, 0.004)	0.65	-0.001 (-0.005, 0.003)	0.61
HMO-bound sialic acid	0.003 (-0.041, 0.047)	0.014 (-0.005, 0.032)	0.67	-0.000 (-0.014, 0.014)	0.99
HMO-bound fucose	0.004 (-0.024, 0.032)	0.004 (-0.007, 0.016)	0.98	0.009 (0.001, 0.018)	**0.043**
2'FL	-0.132 (-0.215, -0.050)	-0.015 (-0.051, 0.020)	**0.001**	0.010 (-0.016, 0.037)	0.45
3FL	0.044 (-0.041, 0.128)	0.007 (-0.029, 0.043)	0.42	0.016 (-0.012, 0.043)	0.26
LNnT	0.013 (-0.059, 0.086)	0.005 (-0.026, 0.036)	0.83	0.005 (-0.029, 0.018)	0.65
3'SL	0.032 (-0.051, 0.115)	0.022 (-0.013, 0.058)	0.84	0.012 (-0.015, 0.038)	0.40
DFLac	0.086 (-0.032, 0.204)	0.028 (-0.022, 0.078)	0.37	0.027 (-0.011, 0.064)	0.17
6'SL	-0.051 (-0.146, 0.045)	0.001 (-0.040, 0.042)	0.33	-0.003 (-0.034, 0.028)	0.84
LNT	0.009 (-0.081, 0.098)	-0.005 (-0.043, 0.033)	0.79	-0.026 (-0.054, 0.003)	0.079
LNFP I	-0.065 (-0.185, 0.055)	-0.012 (-0.063, 0.040)	0.42	-0.018 (-0.056, 0.020)	0.36
LNFP II	-0.015 (-0.089, 0.060)	0.028 (-0.003, 0.060)	0.30	0.002 (-0.022, 0.026)	0.87
LNFP III	0.025 (-0.064, 0.115)	-0.010 (-0.048, 0.028)	0.48	0.007 (-0.022, 0.035)	0.64
LSTb	0.004 (-0.085, 0.092)	0.030 (-0.008, 0.068)	0.59	0.009 (-0.020, 0.037)	0.56
LSTc	0.039 (-0.059, 0.138)	-0.006 (-0.048, 0.036)	0.40	0.001 (-0.031, 0.032)	0.98
DFLNT	-0.017 (-0.160, 0.126)	0.071 (0.010, 0.131)	0.26	0.017 (-0.029, 0.063)	0.46
LNH	0.006 (-0.110, 0.121)	-0.008 (-0.057, 0.041)	0.83	-0.003 (-0.040, 0.034)	0.88
DSLNT	0.037 (-0.055, 0.129)	0.019 (-0.020, 0.058)	0.72	-0.003 (-0.032, 0.026)	0.83
FLNH	-0.103 (-0.236, -0.029)	0.005 (-0.051, 0.061)	0.14	-0.015 (-0.057, 0.028)	0.50
DFLNH	-0.016 (-0.115, 0.083)	0.016 (-0.027, 0.058)	0.56	0.001 (-0.030, 0.033)	0.94
FDSLNH	0.038 (-0.064, 0.140)	0.011 (-0.032, 0.055)	0.63	-0.004 (-0.036, 0.029)	0.83
DSLNH	0.054 (-0.049, 0.157)	-0.025 (-0.068, 0.020)	0.17	-0.028 (-0.061, 0.005)	0.097
Small HMOs	-0.047 (-0.097, 0.002)	-0.009 (-0.030, 0.012)	0.16	-0.006 (-0.022, 0.010)	0.47
Type 1 HMOs	0.028 (-0.038, 0.095)	-0.006 (-0.034, 0.022)	0.35	-0.015 (-0.036, 0.006)	0.15

Type 2 HMOs	0.022 (-0.041, 0.084)	0.002 (-0.025, 0.028)	0.56	-0.002 (-0.022, 0.018)	0.81
α-1-2-fucosylated HMOs	-0.085 (-0.155, -0.015)	-0.018 (-0.047, 0.012)	0.079	0.001 (-0.021, 0.023)	0.94
Terminal α-2-6-sialylated HMOs	-0.045 (-0.128, 0.038)	-0.002 (-0.034, 0.037)	0.30	-0.002 (-0.028, 0.025)	0.89
Internal α-2-6-sialylated HMOs	0.030 (-0.056, 0.115)	0.021 (-0.016, 0.057)	0.85	-0.001 (-0.028, 0.026)	0.94
Terminal α-2-3-sialylated HMOs	0.037 (-0.023, 0.098)	0.019 (-0.006, 0.045)	0.59	0.007 (-0.012, 0.026)	0.47

Supplementary table 8b. Vegetation Cover Diversity, VCDI, and interactions with secretor status and curvilinear associations (**250 m x 250 m grids**). Adjusted log-estimates (95% confidence interval) in HMO diversity, individual HMO concentrations and HMO structural groups (nmol/mL) per one SD increase in greenness separately for non-secretors and secretors from the interaction and from the curvilinear association. Log scale in all other outcome variables expect Diversity, which is in the original scale.

N=760	VCDI*Secretor-interaction			Curvilinear VCDI	
	Non-secretors	Secretors	P-value		P-value
	Est. (95 % CL)	Est. (95 % CL)		Est. (95 % CL)	
Diversity	0.073 (-0.219, 0.364)	0.072 (0.044, 0.189)	0.99	-0.055 (-0.169, 0.060)	0.35
Sum of HMOs	-0.009 (-0.022, 0.005)	-0.001 (-0.007, 0.004)	0.32	-0.003 (-0.002, 0.009)	0.23
HMO-bound sialic acid	-0.010 (-0.057, 0.037)	0.004 (-0.014, 0.023)	0.58	-0.012 (-0.030, 0.007)	0.21
HMO-bound fucose	0.004 (-0.026, 0.033)	0.003 (-0.009, 0.015)	0.96	0.009 (-0.003, 0.020)	0.14
2'FL	-0.175 (-0.263, -0.088)	-0.011 (-0.046, 0.024)	**0.001**	0.039 (0.005, 0.074)	**0.026**
3FL	0.017 (-0.072, 0.105)	0.005 (-0.030, 0.040)	0.80	0.027 (-0.008, 0.061)	0.13
LNnT	0.021 (-0.056, 0.097)	0.004 (-0.027, 0.034)	0.68	0.009 (-0.021, 0.039)	0.57
3'SL	0.014 (-0.074, 0.102)	-0.005 (-0.040, 0.030)	0.70	0.028 (-0.007, 0.062)	0.12
DFLac	0.190 (0.069, 0.312)	0.037 (-0.012, 0.085)	**0.022**	0.002 (-0.047, 0.050)	0.95
6'SL	-0.106 (-0.207, -0.005)	-0.005 (-0.046, 0.036)	0.069	-0.020 (-0.060, 0.020)	0.33
LNT	0.018 (-0.076, 0.113)	0.016 (-0.021, 0.053)	0.97	-0.019 (-0.056, 0.018)	0.31
LNFP I	-0.129 (-0.254, -0.004)	0.000 (-0.050, 0.050)	0.061	0.013 (-0.036, 0.062)	0.60
LNFP II	-0.030 (-0.109, 0.048)	0.024 (-0.008, 0.055)	0.21	-0.012 (-0.043, 0.019)	0.44
LNFP III	-0.001 (-0.095, 0.093)	-0.003 (-0.040, 0.035)	0.98	-0.018 (-0.055, 0.019)	0.33
LSTb	-0.003 (-0.096, 0.091)	0.051 (0.014, 0.088)	0.30	-0.016 (-0.053, 0.020)	0.38
LSTc	0.100 (-0.004, 0.204)	0.003 (-0.038, 0.045)	0.090	0.013 (-0.028, 0.054)	0.52
DFLNT	0.009 (-0.143, 0.161)	0.031 (-0.029, 0.092)	0.79	0.007 (-0.053, 0.067)	0.81
LNH	0.057 (-0.065, 0.179)	-0.009 (-0.057, 0.040)	0.33	0.004 (-0.044, 0.052)	0.86
DSLNT	0.008 (-0.089, 0.106)	0.031 (-0.007, 0.070)	0.66	-0.046 (-0.084, 0.008)	**0.018**
FLNH	-0.016 (-0.16, 0.124)	-0.002 (-0.058, 0.053)	0.86	-0.028 (-0.083, 0.027)	0.32
DFLNH	-0.011 (-0.115, 0.093)	0.009 (-0.032, 0.050)	0.72	-0.004 (-0.045, 0.037)	0.85
FDSLNH	0.080 (-0.028, 0.188)	0.008 (-0.036, 0.050)	0.22	-0.002 (-0.045, 0.040)	0.92
DSLNH	0.118 (0.009, 0.227)	-0.031 (-0.074, -0.013)	**0.013**	0.010 (-0.032, 0.054)	0.62
Small HMOs	-0.082 (-0.134, -0.029)	-0.009 (-0.030, 0.012)	**0.012**	0.012 (-0.008, 0.033)	0.24
Type 1 HMOs	0.013 (-0.056, 0.082)	0.010 (-0.018, 0.037)	0.93	-0.012 (-0.038, 0.016)	0.42

Type 2 HMOs	0.027 (-0.039, 0.092)	0.002 (-0.024, 0.028)	0.49	0.006 (-0.020, 0.032)	0.66
α-1-2-fucosylated HMOs	-0.119 (-0.192, -0.046)	-0.012 (-0.042, 0.017)	**0.009**	0.028 (-0.001, 0.057)	0.055
Terminal α-2-6-sialylated HMOs	-0.091 (-0.179, -0.004)	-0.005 (-0.040, 0.030)	0.072	-0.017 (-0.051, 0.018)	0.35
Internal α-2-6-sialylated HMOs	0.006 (-0.084, 0.096)	0.036 (-0.000, 0.072)	0.54	-0.038 (-0.073, -0.003)	**0.036**
Terminal α-2-3-sialylated HMOs	0.017 (-0.047, 0.082)	0.004 (-0.022, 0.030)	0.70	0.001 (-0.025, 0.026)	0.98

Supplementary table 8c. Naturalness Index, NI, and interactions with secretor status and curvilinear associations (**250 m x 250 m grids**). Adjusted log-estimates (95% confidence interval) in HMO diversity, individual HMO concentrations and HMO structural groups (nmol/mL) per one SD increase in physical living environment (exposure) variables separately for non-secretors and secretors from the interaction and from the curvilinear association. Log scale in all other outcome variables expect Diversity, which is in the original scale.

N=772	NI*Secretor-interaction			Curvilinear NI	
	Non-secretors	Secretors	P-value		P-value
	Est. (95 % CL)	Est. (95 % CL)		Est. (95 % CL)	
Diversity	0.106 (-0.196, 0.407)	0.116 (0.002, 0.230)	0.95	0.007 (-0.071, 0.086)	0.85
Sum of HMOs	-0.006 (-0.021, 0.008)	-0.003 (-0.008, 0.002)	0.65	0.001 (-0.003, 0.004)	0.82
HMO-bound sialic acid	0.010 (-0.038, 0.059)	0.014 (-0.005, 0.032)	0.89	0.005 (-0.007, 0.018)	0.42
HMO-bound fucose	0.016 (-0.015, 0.046)	0.001 (-0.011, 0.012)	0.35	0.002 (-0.006, 0.010)	0.61
2'FL	0.043 (-0.049, 0.134)	-0.029 (-0.063, 0.006)	0.15	-0.001 (-0.025, 0.023)	0.95
3FL	0.079 (-0.013, 0.172)	0.001 (-0.034, 0.036)	0.12	0.019 (-0.005, 0.043)	0.12
LNnT	0.017 (-0.063, 0.097)	0.010 (-0.021, 0.040)	0.86	-0.014 (-0.035, 0.006)	0.18
3'SL	0.041 (-0.051, 0.132)	0.009 (-0.025, 0.044)	0.53	0.025 (0.001, 0.048)	**0.043**
DFLac	0.021 (-0.109, 0.151)	0.021 (-0.028, 0.070)	0.99	0.031 (-0.003, 0.064)	0.076
6'SL	-0.025 (-0.130, 0.081)	0.008 (-0.032, 0.047)	0.57	-0.007 (-0.034, 0.021)	0.64
LNT	-0.051 (-0.149, 0.048)	0.012 (-0.025, 0.050)	0.24	-0.013 (-0.038, 0.013)	0.33
LNFP I	-0.175 (-0.306, 0.044)	-0.004 (-0.053, 0.046)	**0.017**	-0.011 (-0.045, 0.022)	0.53
LNFP II	0.029 (-0.053, 0.110)	0.017 (-0.014, 0.048)	0.80	-0.008 (-0.029, 0.013)	0.45
LNFP III	0.017 (-0.081, 0.115)	-0.017 (-0.054, 0.020)	0.52	-0.006 (-0.031, 0.020)	0.67
LSTb	-0.015 (-0.112, 0.082)	0.054 (0.017, 0.091)	0.19	-0.003 (-0.028, 0.022)	0.82
LSTc	0.107 (-0.001, 0.215)	-0.044 (-0.085, -0.003)	**0.010**	0.010 (-0.018, 0.038)	0.49
DFLNT	0.034 (-0.123, 0.191)	0.055 (-0.004, 0.114)	0.81	0.008 (-0.033, 0.049)	0.70
LNH	-0.013 (-0.139, 0.114)	-0.023 (-0.071, 0.025)	0.88	-0.001 (-0.034, 0.032)	0.95
DSLNT	-0.010 (-0.111, 0.091)	0.035 (0.003, 0.073)	0.41	0.003 (-0.023, 0.029)	0.81
FLNH	-0.126 (-0.272, -0.019)	0.004 (-0.059, 0.050)	0.12	0.015 (-0.023, 0.052)	0.45
DFLNH	-0.037 (-0.146, 0.072)	-0.018 (-0.059, 0.023)	0.75	-0.026 (-0.055, 0.002)	0.067
FDSLNH	0.101 (-0.011, 0.213)	-0.001 (-0.043, 0.042)	0.10	-0.018 (-0.048, 0.011)	0.22
DSLNH	0.114 (0.001, 0.227)	-0.050 (-0.092, -0.007)	**0.008**	0.001 (-0.029, 0.030)	0.99
Small HMOs	-0.082 (-0.134, -0.029)	-0.009 (-0.030, 0.012)	**0.012**	0.006 (-0.009, 0.020)	0.43
Type 1 HMOs	0.013 (-0.056, 0.082)	0.010 (-0.018, 0.037)	0.93	-0.010 (-0.029, 0.009)	0.28

Type 2 HMOs	0.027 (-0.039, 0.092)	0.002 (-0.024, 0.028)	0.49	-0.012 (-0.029, 0.006)	0.21
α-1-2-fucosylated HMOs	-0.119 (-0.192, -0.045)	-0.012 (-0.042, 0.017)	**0.009**	-0.001 (-0.020, 0.020)	0.99
Terminal α-2-6-sialylated HMOs	-0.091 (-0.179, -0.004)	-0.005 (-0.040, 0.030)	0.072	-0.004 (-0.027, 0.020)	0.77
Internal α-2-6-sialylated HMOs	0.006 (-0.084, 0.096)	0.036 (-0.000, 0.072)	0.54	0.003 (-0.021, 0.027)	0.81
Terminal α-2-3-sialylated HMOs	0.017 (-0.047, 0.082)	0.004 (-0.022, 0.030)	0.70	0.018 (0.001, 0.035)	**0.041**

Supplementary table 9a. Socioeconomic disadvantage (750 m x 750 m grids) adjusted standardized estimates (95% confidence interval) in HMO diversity, individual HMO concentrations and HMO structural groups (nmol/mL) per one SD increase in physical living environment (exposure) variables. Standardization means that both the exposure and outcome have been standardized (log scale in all other outcome variables expect Diversity, which is in the original scale). In addition to the confounders included in the main models (Table 2) these models have been adjusted with residential socioeconomic disadvantage. Statistics are given for main effects. * indicates a significant interaction between the exposure variable and secretor status (p<0.05). [C] indicates a significant curvilinear association with the exposure variable (p<0.05).

N=683	NDVI, Normalized Difference Vegetation Index		VCDI, Vegetation Cover Diversity		NI, Naturalness Index	
	Est. (95 % CL)	p-value	Est. (95 % CL)	p-value	Est. (95 % CL)	p-value
Diversity	0.037 (-0.040, 0.115)	0.35	0.086 (0.010, 0.162)	**0.026**[C]	0.099 (0.016, 0.181)	**0.019**
Sum of HMOs	-0.005 (-0.034, 0.019)	0.65	-0.019 (-0.044, 0.010)	0.16	-0.029 (-0.053, 0.005)	0.072
HMO-bound sialic acid	0.018 (-0.049, 0.085)	0.58	0.018 (-0.049, 0.085)	0.61	0.035 (-0.039, 0.106)	0.36
HMO-bound fucose	0.005 (-0.026, 0.037)	0.72	-0.010 (-0.042, 0.018)	0.46	-0.005 (-0.037, 0.029)	0.78
2'FL	-0.030 (-0.051, -0.008)	**0.007***	-0.031 (-0.052, -0.010)	**0.005***	-0.031 (-0.054, -0.008)	**0.009***
3FL	-0.014 (-0.072, 0.043)	0.64	-0.019 (-0.075, 0.038)	0.52	-0.043 (-0.104, 0.018)	0.16
LNnT	0.077 (-0.002, 0.154)	0.051	0.072 (-0.005, 0.147)	0.066	0.094 (0.012, 0.176)	**0.025**
3'SL	-0.032 (-0.106, 0.044)	0.42	-0.032 (-0.106, 0.040)	0.38	-0.068 (-0.148, 0.010)	0.093
DFLac	0.017 (-0.015, 0.048)	0.30*	0.030 (-0.001, 0.061)	0.061*	0.015 (-0.019, 0.048)	0.39
6'SL	-0.002 (-0.074, 0.071)	0.96	-0.014 (-0.083, 0.057)	0.72*	0.026 (-0.052, 0.102)	0.51*
LNT	0.016 (-0.061, 0.093)	0.69	0.093 (0.018, 0.168)	**0.015**	0.075 (-0.008, 0.158)	0.074
LNFP I	0.006 (-0.039, 0.050)	0.80	0.011 (-0.033, 0.054)	0.63	-0.008 (-0.039, 0.055)	0.73*
LNFP II	0.057 (-0.013, 0.126)	0.11	0.038 (-0.030, 0.106)	0.26	0.057 (-0.017, 0.132)	0.13
LNFP III	-0.006 (-0.075, 0.066)	0.89	0.011 (-0.059, 0.081)	0.77	-0.018 (-0.094, 0.059)	0.64
LSTb	0.064 (-0.010, 0.137)	0.090	0.114 (0.042, 0.185)	**0.002**	0.121 (0.044, 0.198)	**0.002***
LSTc	0.018 (-0.058, 0.092)	0.66	-0.012 (-0.086, 0.060)	0.73	-0.005 (-0.085, 0.076)	0.90
DFLNT	0.074 (-0.001, 0.143)	0.053	0.051 (-0.023, 0.124)	0.17	0.093 (0.013, 0.172)	**0.023**
LNH	0.006 (-0.073, 0.087)	0.87	0.036 (-0.043, 0.114)	0.37	-0.002 (-0.087, 0.084)	0.97
DSLNT	0.070 (-0.006, 0.148)	0.068	0.098 (0.023, 0.172)	**0.011**	0.107 (0.025, 0.189)	**0.010**
FLNH	-0.025 (-0.10, 0.050)	0.52	0.040 (-0.034, 0.112)	0.29	0.012 (-0.069, 0.092)	0.77*
DFLNH	0.040 (-0.026, 0.104)	0.24	0.034 (-0.031, 0.098)	0.31	0.060 (-0.009, 0.130)	0.091
FDSLNH	0.034 (-0.036, 0.103)	0.33	0.041 (-0.028, 0.108)	0.24	0.023 (-0.050, 0.097)	0.53
DSLNH	-0.010 (-0.087, 0.066)	0.79	-0.060 (-0.133, 0.015)	0.12	-0.009 (-0.090, 0.073)	0.83*

Small HMOs	-0.039 (-0.076, -0.003)	**0.032**[C]	-0.056 (-0.092, -0.022)	**0.002***	-0.068 (-0.105, -0.029)	**0.001***
Type 1 HMOs	0.050 (-0.019, 0.119)	0.16	0.083 (0.002, 0.150)	**0.013**	0.081 (0.007, 0.153)	**0.031**
Type 2 HMOs	0.072 (-0.006, 0.147)	0.068	0.069 (-0.006, 0.147)	0.069	0.086 (0.003, 0.167)	**0.042**
α-1-2-fucosylated HMOs	-0.021 (-0.043, 0.001)	0.053*	-0.022 (-0.043, -0.001)	**0.042**	-0.030 (-0.053, -0.007)	**0.012***
Terminal α-2-6-sialylated HMOs	0.004 (-0.076, 0.066)	0.89	-0.016 (-0.088, 0.054)	0.64*	0.022 (-0.056, 0.098)	0.58*
Internal α-2-6-sialylated HMOs	0.077 (0.002, 0.151)	**0.047**	0.106 (0.031, 0.178)	**0.005**	0.114 (0.035, 0.195)	**0.005**
Terminal α-2-3-sialylated HMOs	0.003 (-0.070, 0.079)	0.92	0.015 (-0.058, 0.087)	0.70	-0.017 (-0.096, 0.064)	0.69

[1]interaction p-value 0.055

Supplementary table 9b. Socioeconomic disadvantage (750 m x 750 m grids) adjusted log-estimates (95% confidence interval), prior to outcome standardization, in HMO diversity, individual HMO concentrations and HMO structural groups (nmol/mL) per one SD increase in physical living environment (exposure) variables. Log scale in all other outcome variables expect Diversity, which is in the original scale. In addition to the confounders included in the main models (Table 2) these models have been adjusted with residential socioeconomic disadvantage. Statistics are given for main effects. * indicates a significant interaction between the exposure variable and secretor status ($p<0.05$). ^{C} indicates a significant curvilinear association with the exposure variable ($p<0.05$).

N=683	NDVI, Normalized Difference Vegetation Index		VCDI, Vegetation Cover Diversity		NI, Naturalness Index	
	Est. (95 % CL)	p-value	Est. (95 % CL)	p-value	Est. (95 % CL)	p-value
Diversity	0.057 (-0.062, 0.176)	0.35	0.132 (0.016, 0.248)	**0.026**^{C}	0.152 (0.025, 0.278)	**0.019**
Sum of HMOs	-0.001 (-0.007, 0.004)	0.65	-0.004 (-0.009, 0.002)	0.16	-0.006 (-0.011, 0.001)	0.072
HMO-bound sialic acid	0.005 (-0.014, 0.024)	0.58	0.005 (-0.014, 0.024)	0.61	0.010 (-0.011, 0.030)	0.36
HMO-bound fucose	0.002 (-0.010, 0.014)	0.72	-0.004 (-0.016, 0.007)	0.46	-0.002 (-0.014, 0.011)	0.78
2'FL	-0.050 (-0.086, -0.014)	**0.007***	-0.052 (-0.087, -0.016)	**0.005***	-0.052 (-0.090, -0.013)	**0.009***
3FL	-0.009 (-0.045, 0.027)	0.64	-0.012 (-0.047, 0.024)	0.52	-0.027 (-0.065, 0.011)	0.16
LNnT	0.031 (-0.001, 0.062)	0.051	0.029 (-0.002, 0.059)	0.066	0.038 (0.005, 0.071)	**0.025**
3'SL	-0.015 (-0.050, 0.021)	0.42	-0.015 (-0.050, 0.019)	0.38	-0.032 (-0.070, 0.005)	0.093
DFLac	0.027 (-0.024, 0.078)	0.30*	0.048 (-0.002, 0.098)	0.061*	0.024 (-0.031, 0.078)	0.39
6'SL	-0.001 (-0.043, 0.041)	0.96	-0.008 (-0.048, 0.033)	0.72*	0.015 (-0.030, 0.059)	0.51*
LNT	0.008 (-0.031, 0.047)	0.69	0.047 (0.009, 0.085)	**0.015**	0.038 (-0.004, 0.080)	0.074
LNFP I	0.007 (-0.046, 0.059)	0.80	0.013 (-0.039, 0.064)	0.63	0.010 (-0.046, 0.066)	0.73*
LNFP II	0.027 (-0.006, 0.059)	0.11	0.018 (-0.014, 0.050)	0.26	0.027 (-0.008, 0.062)	0.13
LNFP III	-0.003 (-0.041, 0.036)	0.89	0.006 (-0.032, 0.044)	0.77	-0.010 (-0.051, 0.032)	0.64
LSTb	0.033 (-0.005, 0.071)	0.09	0.059 (0.022, 0.096)	**0.002**	0.063 (0.023, 0.103)	**0.002***
LSTc	0.010 (-0.033, 0.052)	0.66	-0.007 (-0.049, 0.034)	0.73	-0.003 (-0.048, 0.043)	0.90
DFLNT	0.062 (-0.001, 0.120)	0.053	0.043 (-0.019, 0.104)	0.17	0.078 (0.011, 0.144)	**0.023**
LNH	0.004 (-0.046, 0.055)	0.87	0.023 (-0.027, 0.072)	0.37	-0.001 (-0.055, 0.053)	0.97
DSLNT	0.036 (-0.003, 0.076)	0.068	0.050 (0.012, 0.088)	**0.011**	0.055 (0.013, 0.097)	**0.010**
FLNH	-0.019 (-0.077, 0.039)	0.52	0.031 (-0.026, 0.087)	0.29	0.009 (-0.053, 0.071)	0.77*
DFLNH	0.026 (-0.017, 0.068)	0.24	0.022 (-0.020, 0.064)	0.31	0.039 (-0.006, 0.085)	0.091
FDSLNH	0.022 (-0.023, 0.066)	0.33	0.026 (-0.018, 0.069)	0.24	0.015 (-0.032, 0.062)	0.53
DSLNH	-0.006 (-0.051, 0.039)	0.79	-0.035 (-0.078, 0.009)	0.12	-0.005 (-0.053, 0.043)	0.83*
Small HMOs	-0.023 (-0.045, -0.002)	**0.032**^{C}	-0.033 (-0.054, -0.012)	**0.002***	-0.040 (-0.062, -0.017)	**0.001***

Type 1 HMOs	0.021 (-0.008, 0.050)	0.16	0.035 (0.0008, 0.063)	**0.013**	0.034 (0.003, 0.064)	**0.031**
Type 2 HMOs	0.025 (-0.002, 0.051)	0.068	0.024 (-0.002, 0.051)	0.069	0.030 (0.001, 0.058)	**0.042**
α-1-2-fucosylated HMOs	-0.030 (-0.060, 0.001)	0.053*	-0.031 (-0.061, -0.001)	**0.042**	-0.042 (-0.074, -0.010)	**0.012***
Terminal α-2-6-sialylated HMOs	0.002 (-0.038, 0.033)	0.89	-0.008 (-0.044, 0.027)	0.64*	0.011 (-0.028, 0.049)	0.58
Internal α-2-6-sialylated HMOs	0.037 (0.001, 0.073)	**0.047**	0.051 (0.015, 0.086)	**0.005**	0.055 (0.017, 0.094)	**0.005**
Terminal α-2-3-sialylated HMOs	0.001 (-0.024, 0.027)	0.92	0.005 (-0.020, 0.030)	0.70	-0.006 (-0.033, 0.022)	0.69

Supplementary figure 1a. Associations between greenness, NDVI, and HMO components from adjusted models in the original scale (750 m x 750 m grids). Only statistically significant associations (main, interaction or curvilinear) are shown separately for non-secretors and secretors. When the scales differ vastly between the groups, separate y-axes are given for non-secretors (blue) and secretors (red).

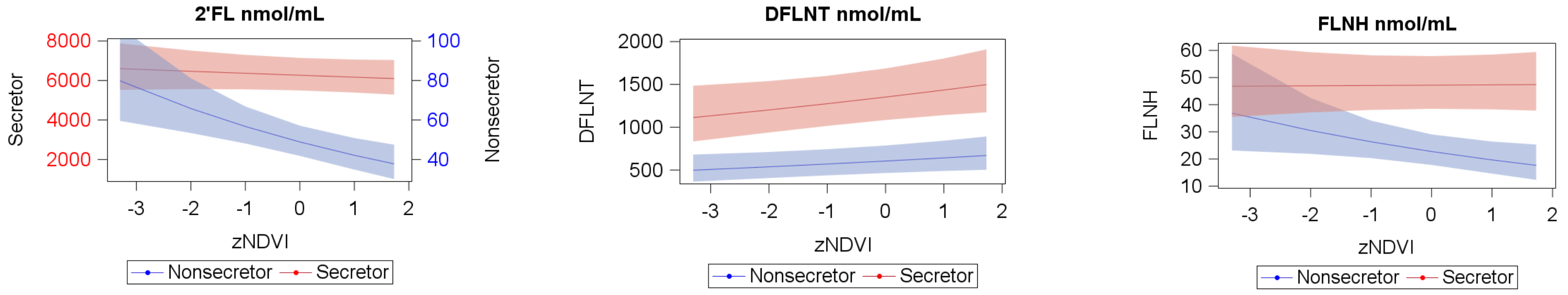

Supplementary figure 1b. Associations between vegetation cover diversity, VCDI, and HMO components from adjusted models in the original scale (750 m x 750 m grids). Only statistically significant associations (main, interaction or curvilinear) are shown separately for non-secretors and secretors. When the scales differ vastly between the groups, separate y-axes are given for non-secretors (blue) and secretors (red).

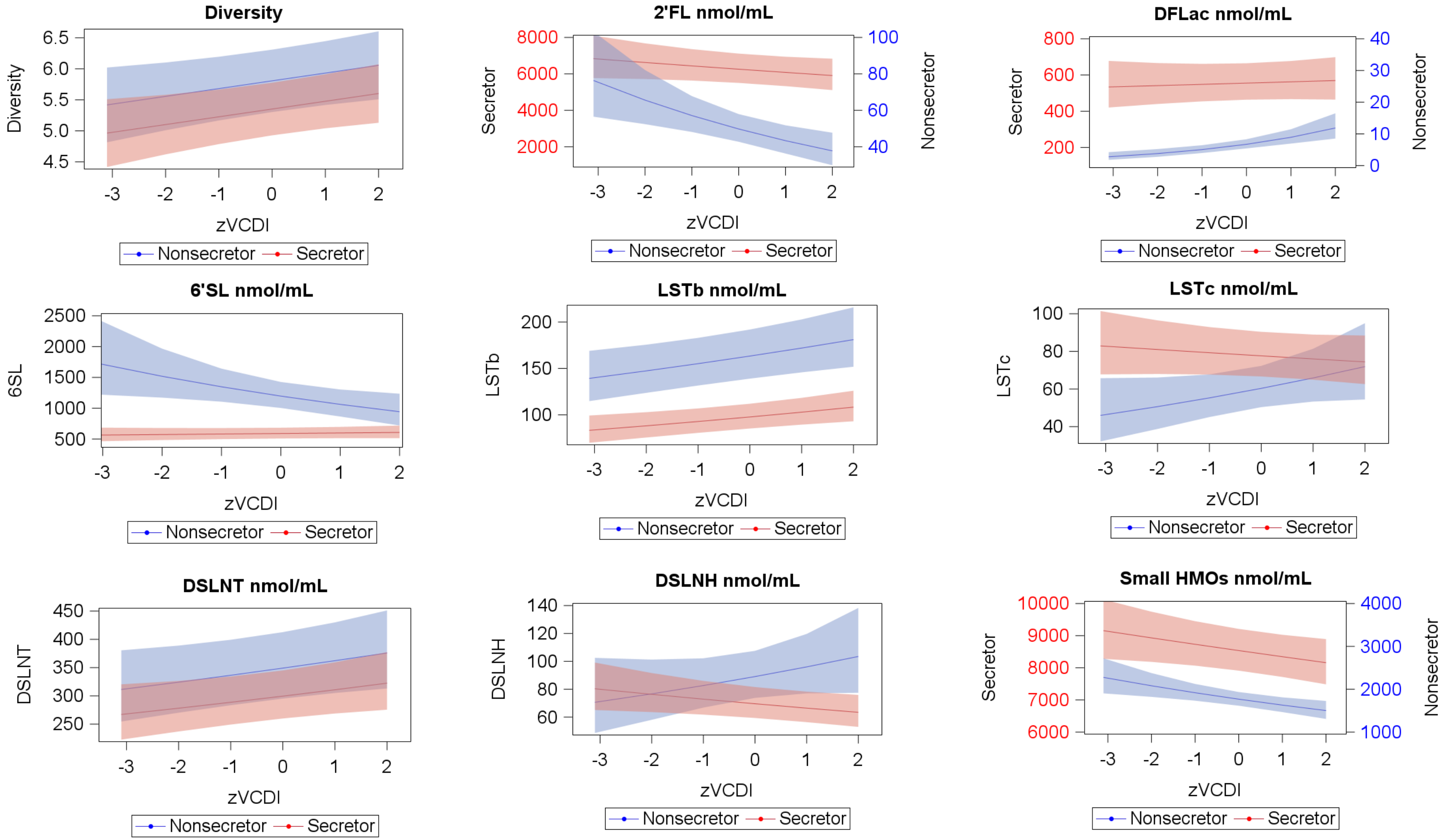

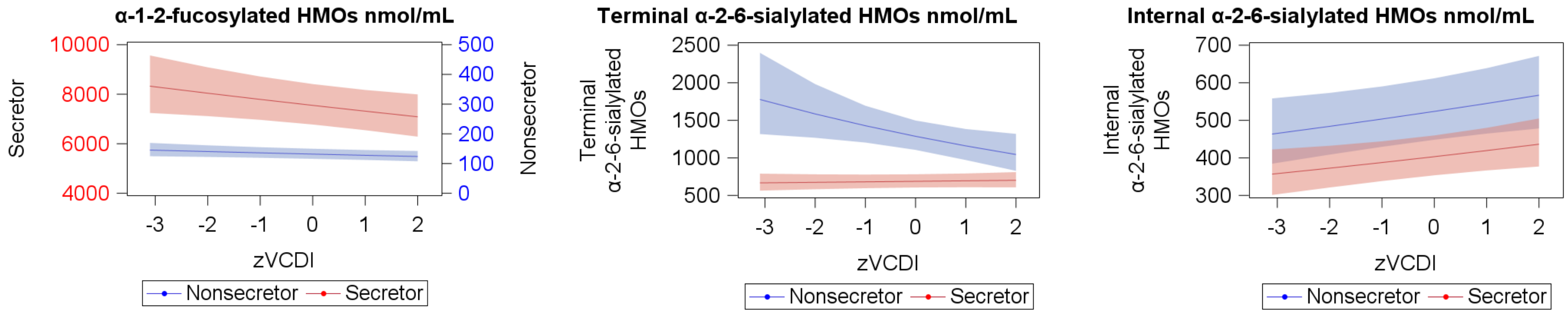
α-1-2-fucosylated HMOs nmol/mL
Secretor
Nonsecretor
10000
8000
6000
4000
500
400
300
200
100
0
-3
-2
-1
0
1
2
zVCDI
Nonsecretor
Secretor
Terminal α-2-6-sialylated HMOs nmol/mL
Terminal α-2-6-sialylated HMOs
2500
2000
1500
1000
500
zVCDI
Nonsecretor
Secretor
Internal α-2-6-sialylated HMOs nmol/mL
Internal α-2-6-sialylated HMOs
700
600
500
400
300
zVCDI
Nonsecretor
Secretor

Supplementary figure 1c. Associations between naturalness, NI, and HMO components from adjusted models in the original scale (750 m x 750 m grids). Only statistically significant associations (main, interaction or curvilinear) are shown separately for non-secretors and secretors. When the scales differ vastly between the groups, separate y-axes are given for non-secretors (blue) and secretors (red).

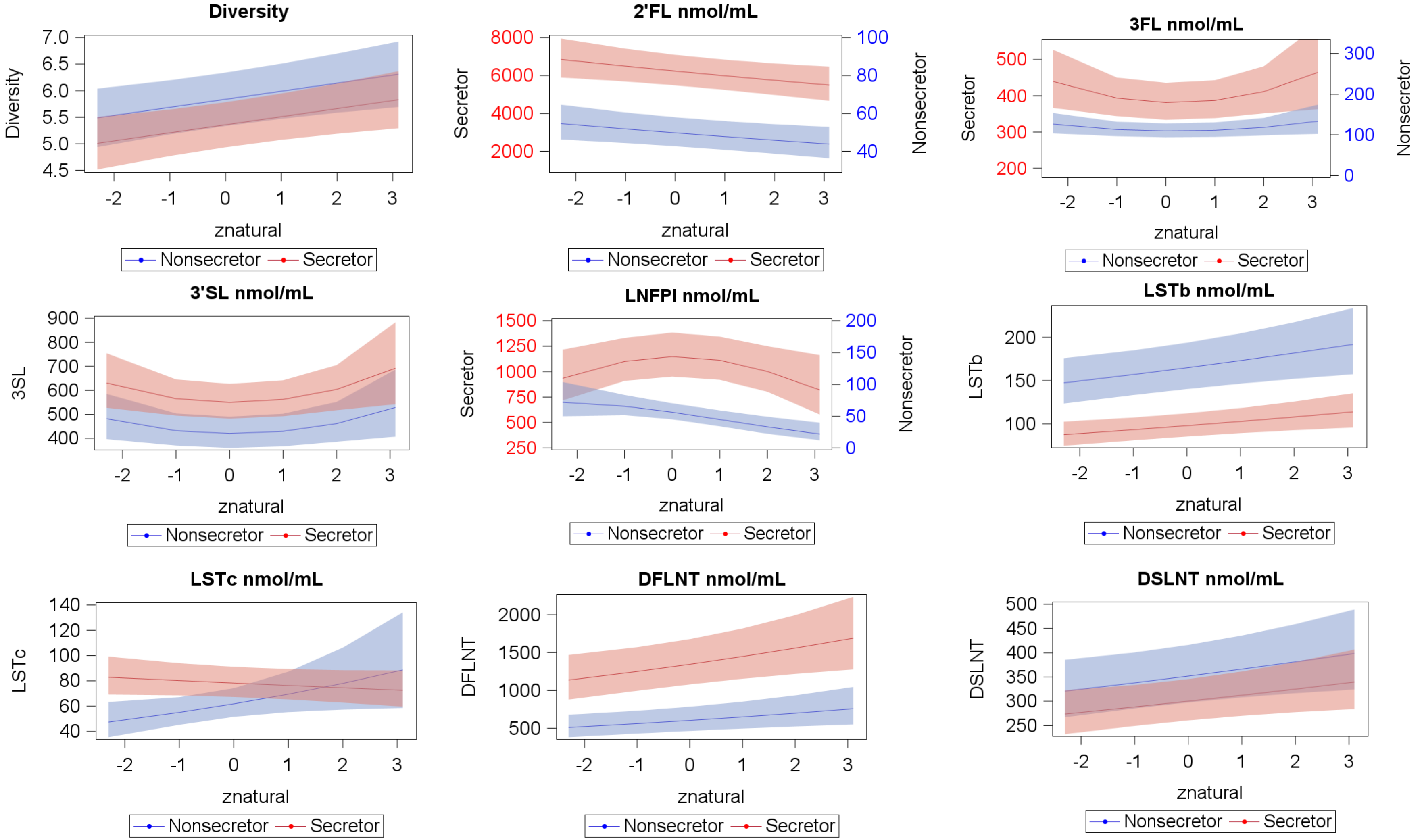

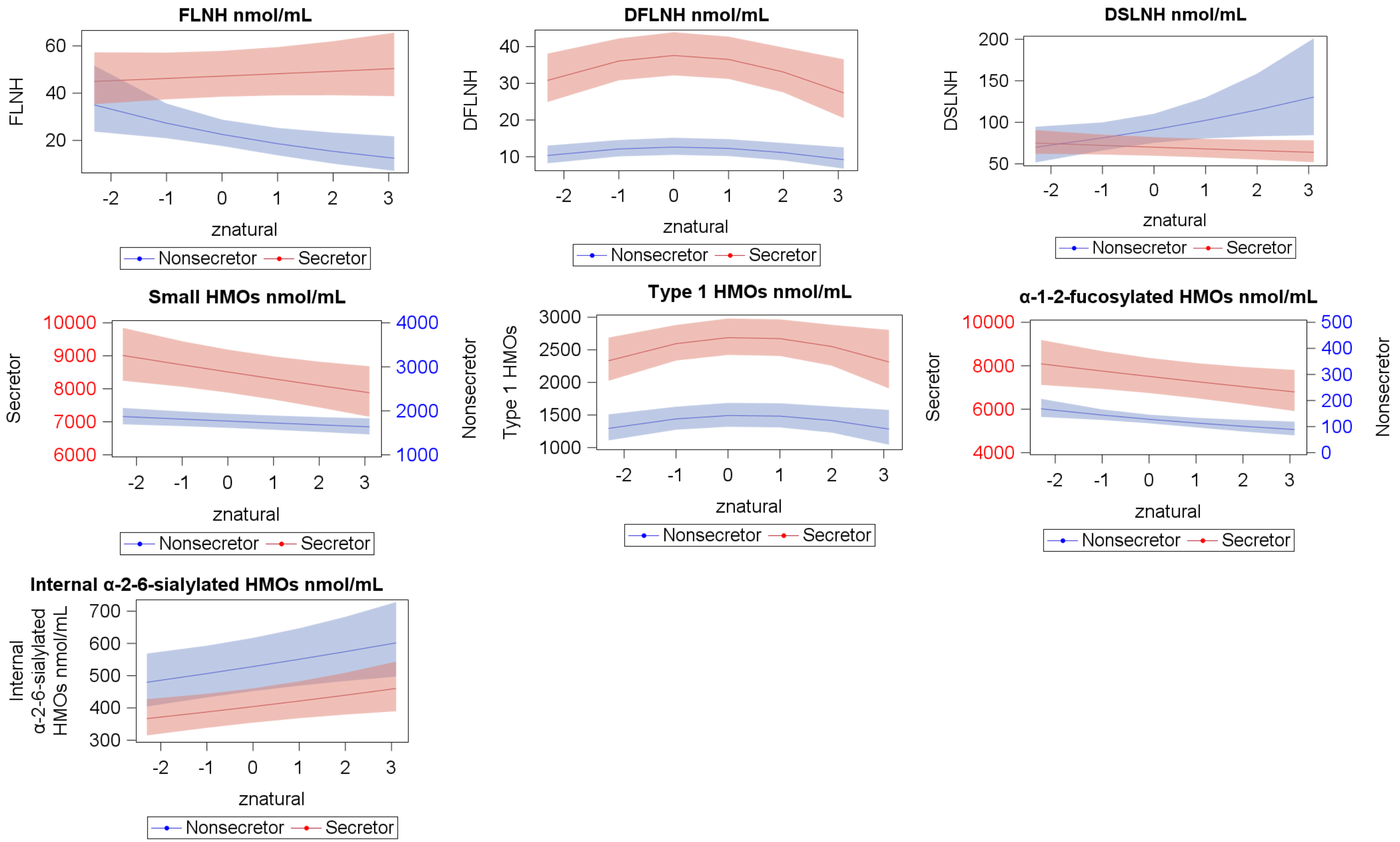
FLNH nmol/mL
FLNH
60
40
20
-2 -1 0 1 2 3
znatural
Nonsecretor
Secretor
DFLNH nmol/mL
DFLNH
40
30
20
10
-2 -1 0 1 2 3
znatural
Nonsecretor
Secretor
DSLNH nmol/mL
DSLNH
200
150
100
50
-2 -1 0 1 2 3
znatural
Nonsecretor
Secretor
Small HMOs nmol/mL
Secretor
10000
9000
8000
7000
6000
Nonsecretor
4000
3000
2000
1000
-2 -1 0 1 2 3
znatural
Nonsecretor
Secretor
Type 1 HMOs nmol/mL
Type 1 HMOs
3000
2500
2000
1500
1000
-2 -1 0 1 2 3
znatural
Nonsecretor
Secretor
α-1-2-fucosylated HMOs nmol/mL
Secretor
10000
8000
6000
4000
Nonsecretor
500
400
300
200
100
0
-2 -1 0 1 2 3
znatural
Nonsecretor
Secretor
Internal α-2-6-sialylated HMOs nmol/mL
Internal α-2-6-sialylated HMOs nmol/mL
700
600
500
400
300
-2 -1 0 1 2 3
znatural
Nonsecretor
Secretor

Supplementary figure 2. Flow chart of the study population included in the analyses.

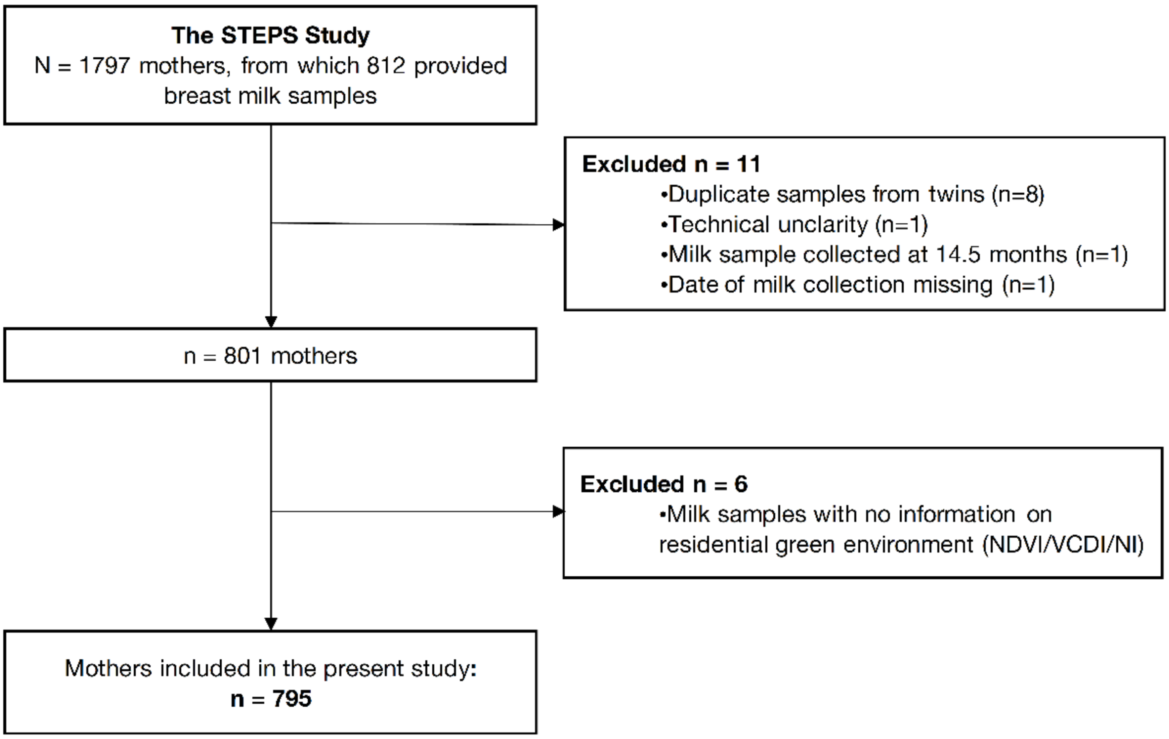

Supplement: Supplementary file 1 — Supplementary Information. [file 41598_2022_27317_MOESM1_ESM.pdf]
